# Engineered Decellularized Matrix Hydrogels with Crypt–Villus Topography for Forming Functional Intestinal Epithelium

**DOI:** 10.1002/smll.202506632

**Published:** 2025-10-22

**Authors:** Ngoc Ha Luong, Van Thuy Duong, Jonathan B. Bryan, Chien‐Chi Lin

**Affiliations:** ^1^ Weldon School of Biomedical Engineering Purdue University West Lafayette IN 47907 USA

**Keywords:** 3D printing, crypt/villus, decellularized matrix, intestinal epithelial barrier, inverse molding, sacrificial hydrogels

## Abstract

Creating a functional intestine model remains challenging owing to the complex topography of the intestinal crypt/villus structure. Here, an inverse molding biofabrication technique is presented to address this challenge. A sacrificial hydrogel mold (SHM) with negative crypt/villus features is first fabricated using digital light processing (DLP) 3D printing of poly(ethylene glycol)–norbornene–tyramine (PEGNB‐T) thiol–norbornene hydrogels. Next, decellularized small intestine submucosa–norbornene (dSIS–NB) solution is cast and photopolymerized over the SHM, also via efficient thiol–norbornene photoclick reaction using PEG‐tetrathiol as the crosslinker. The hydrogel construct is placed in a buffer solution to induce autonomous and rapid dissolution of the SHM, creating dSIS–NB hydrogels with the positive crypt/villus structure. Intestinal epithelial cells seeded on the dSIS–NB crypt/villus matrices form a confluent monolayer within 3 days and display correct intestinal polarity. Through transepithelial electrical resistance (TEER) measurements and macromolecular transport studies, the new inverse‐molded dSIS–NB crypt/villus model further demonstrates the selective and drug‐responsive barrier functions. Finally, the unique biofabrication technique is leveraged to create an intestinal disease model carrying regions of both healthy crypts–villi structure and flattened epithelium with hindered macromolecular transport.

## Graphical abstract

A decellularized matrix hydrogel is fabricated with intestinal crypt/villus topography using an inverse‐molding technique over a sacrificial hydrogel with rapid and autonomous dissolution. Integrating a biofabrication workflow with a matrix with proper stiffness, bioactivity, and topography creates a functional intestinal barrier suitable for drug testing and disease modeling.

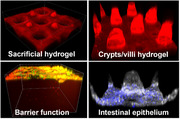

## 1 Introduction

The human small intestine is a highly specialized organ that maintains gut homeostasis by controlling the passage of nutrients and microorganisms.^[^ ^1^, ^2^, ^3^ ^]^ Significant efforts are dedicated to creating substrates/matrices that can replicate the structure and function of intestinal epithelium,^[^ ^4^ ^]^ including two‐dimensional (2D) flat transwell inserts and three‐dimensional (3D) hydrogels.^[^ ^5^, ^6^ ^]^ However, 2D transwell surfaces fail to mimic the intricate intestinal crypt/villus architecture and mechanical properties,^[^ ^7^ ^]^ leading to overly tight cellular junctions, abnormally high transepithelial electrical resistance (TEER), and ultralow permeability.^[^ ^8^, ^9^ ^]^ On the other hand, 3D hydrogels could be leveraged to create human intestinal organoids, but most hydrogel‐based organoid models are limited by their inability to control crypt/villus architecture.^[^ ^10^ ^]^ While engineered photoresponsive hydrogels have been developed to generate intestinal organoids with controlled crypt morphogenesis,^[^ ^11^, ^12^ ^]^ no villus structures were generated. Furthermore, the organoids still had an inverse polarity that was inaccessible to drug transport and absorption.^[^ ^13^, ^14^ ^]^

Traditional microfabrication technology has been utilized to create templates for molding polymer substrates that permit the formation of intestinal epithelium with accessible crypt/villus topography. For example, a polydimethylsiloxane (PDMS)‐based micromolding technique was employed to create a collagen matrix with the crypt/villus structure.^[^ ^15^ ^]^ However, the photoresist‐based microfabrication technique was laborious and not adaptable for most laboratories. Alternatively, the crypt–villus structure could be fabricated and molded using a micromilling machine.^[^ ^16^ ^]^ While this approach did not rely on the cleanroom technology, the intestinal cells were grown on a PDMS membrane that was stiffer than natural intestinal tissue. In this regard, modern 3D printing technology offers a highly adaptable platform for creating intestine‐mimetic substrates. For example, Elomaa et al. modified porcine small intestine submucosa (dSIS) into photocrosslinkable dSIS–methacryloyl (dSIS–MA), which was subsequently 3D‐printed into an array of villi using digital light processing (DLP) 3D printing.^[^ ^17^ ^]^ Notably, the critical crypt structure was missing in this study. Additionally, most protein‐based bioinks have poor printability due to their high viscosity and lack of tunability. As such, synthetic materials are commonly employed to increase the printability of protein‐based bioink, but they would create matrices with nonphysiologically high stiffness.^[^ ^18^, ^19^ ^]^ To address the low printability of protein‐based bioink, Rudolph et al. developed a multistep fabrication process to create silk scaffolds with crypts and villi.^[^ ^20^ ^]^ In this process, a polymer resin with crypt and villus structures was first DLP‐printed, followed by casting an inverse PDMS mold and coating the PDMS mold with a thin silk film. After drying and annealing, the silk‐film‐coated PDMS was finally used to mold the silk scaffold with actual crypts and villi. In addition to the complicated fabrication process, the PDMS mold must be manually removed, which could damage the delicate crypt/villus structure. Furthermore, none of these prior examples demonstrated physiologically relevant molecular transport.

Here, we report a novel two‐step biofabrication approach to create a biomimetic crypt/villus structure. In the first step, a sacrificial hydrogel mold (SHM) was formulated by 3D DLP printing of photocrosslinkable and dissolvable poly(ethylene glycol) (PEG)–norbornene–tyramine (PEGNB‐T) hydrogels.^[^ ^21^ ^]^ Next, the DLP‐printed SHM was directly used to mold cell‐instructive dSIS–norbornene (dSIS–NB) hydrogels without any surface coating process. Both dissolvable SHM hydrogels and cell‐instructive dSIS–NB hydrogels were crosslinked by rapid thiol–norbornene photoclick reaction.^[^ ^22^ ^]^ After these two steps, the construct was inverted and incubated in transwell inserts to allow for rapid and autonomous dissolution of the PEGNB‐T‐based SHM, leaving the cell‐instructive dSIS–NB hydrogel with crypt/villus topography. The biological relevance of the dSIS–NB crypt/villus hydrogels was evaluated via intestinal epithelial cell growth and polarity, macromolecular permeability, and responses to toxic agents. Finally, a proof‐of‐concept intestinal disease model was created using the same biofabrication design principle but with both intact crypt/villus and impaired/flat topographies on the same substrate, permitting side‐by‐side observation and comparison of dysfunction and healthy intestinal tissues.

## 2 Results and Discussion

### 2.1 Suitability of dSIS–NB Hydrogels for Intestinal Epithelialization

**Figure 1:**
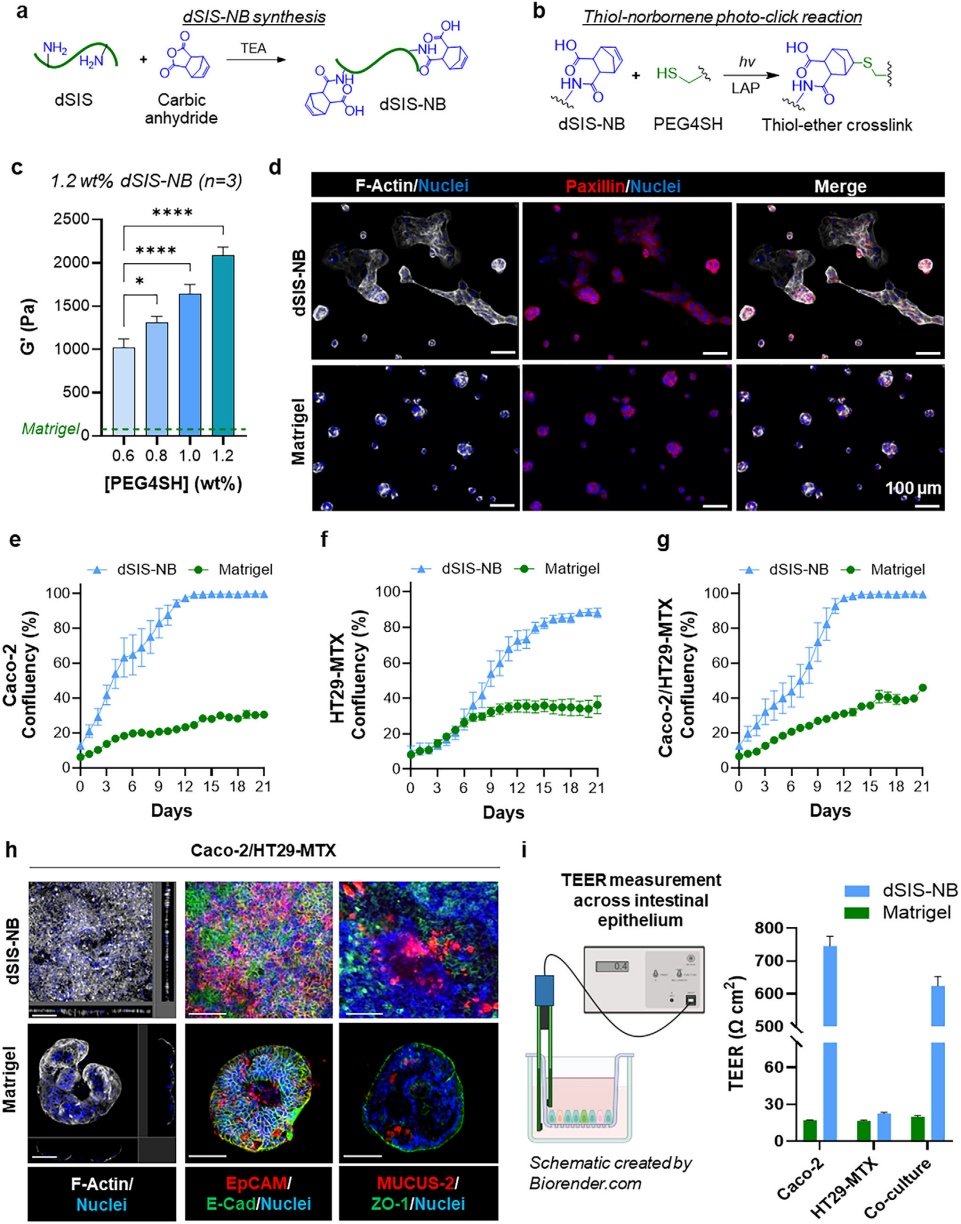
dSIS–NB hydrogel crosslinking and intestinal cell culture. a) Schematic of dSIS–NB synthesis using TEA as a base catalyst. b) Reaction scheme of thiol–norbornene photoclick reaction used for dSIS–NB hydrogel crosslinking. c) Hydrogel stiffness was adjusted by tuning crosslinker (i.e., 4‐arm PEG–thiol or PEG4SH) concentration (i.e., 1.2, 1.0, 0.8, or 0.6 wt%) at fixed dSIS–NB (1.2 wt%) and LAP contents (7 mm). Light wavelength: 365 nm. Intensity: 5 mW cm^−2^; 2 min. Matrigel was used as a control. Data are presented as mean ± SEM (*n* = 3, * and **** represent *p* < 0.05 and 0.0001, respectively, by ordinary one‐way ANOVA with Dunnert's multiple comparisons test, with a single pooled variance). d) Representative immunostaining images of F‐actin (white) and paxillin (red) staining showing differences in protrusions and focal adhesion 1 day post cell seeding on dSIS–NB hydrogel (top) or Matrigel (bottom). Scale bars = 100 µm. e–g) Live cell tracking of intestinal cell growth on thick (≈1.15 mm) and flat dSIS–NB hydrogels or Matrigel: e) Caco‐2, f) HT29‐MTX, and g) Caco‐2/HT29‐MTX (9:1, total cell density of 1 × 10^5^ cells cm^−2^). Data are presented as mean ± SEM (*n* = 4). h) Representative immunostaining images of F‐actin, EpCAM, E‐Cad, Mucin‐2, and ZO‐1 in Caco‐2/HT29‐MTX co‐culture on dSIS–NB hydrogels (top) or Matrigel (bottom) after 21 days of culture. Scale bars = 200 µm. i) TEER values of Caco‐2 and HT29‐MTX monocultures and cocultures on dSIS–NB hydrogel or Matrigel, measured on day 21. TEER values are presented as mean ± SEM (*n* = 2 for Matrigel or 4 for dSIS–NB hydrogels). The schematic was created by Biorender.com.

dSIS–NB was synthesized by reacting dSIS with carbic anhydride (CA; **Figure** **1**a),^[^ ^22^ ^]^ with a degree of NB substitution determined to be ≈10%. Proteomics analysis results show that both dSIS and dSIS–NB exhibited similar protein makeup, with more than 60% of type I collagen (COL1A1 and COL1A2), ≈20% of type III collagen (COL3A1), and ≈15% of fibrillins (FBN1 and FBN2) (Figure S1, Supporting Information). These structural proteins establish a fibrillar matrix that is distinctly different from commonly used matrices such as Matrigel, which is rich in glycoproteins (e.g., laminin) and collagen IV.^[^ ^23^ ^]^ The functionalization with NB did not alter protein composition in dSIS, but afforded thiol–norbornene photocrosslinking (Figure 1b) into hydrogels with physiologically relevant moduli (*G*′ ≈ 1.0–2.1 kPa; Figure 1c).^[^ ^24^, ^25^ ^]^ Chemically crosslinked dSIS–NB hydrogels supported early Caco‐2 cell adhesion on dSIS–NB hydrogels, as revealed by prominent stress fibers and lamellipodia/filopodia‐like protrusions (Figure 1d). In contrast, Caco‐2 cells seeded on Matrigel presented an aggregated morphology with few stress fibers. Interestingly, dSIS–NB hydrogels with shear moduli (*G*′) of ≈1 kPa supported initial cell attachment and proliferation, but the gels degraded after 10 days of culture (Figure S2a, Supporting Information). At the same dSIS–NB concentration (1.2 wt%), increasing crosslinker PEG4SH content led to stiffer dSIS–NB hydrogels (*G*′ ≈ 2 kPa) that supported long‐term stability (over 3 weeks) for cell attachment and proliferation (Figure S2a, Supporting Information). This result aligns with the existing literature on the effects of substrate stiffness on cytoskeleton and focal adhesion dynamics.^[^ ^26^ ^]^ The optimal cell seeding density was evaluated, with 1 × 10^5^ cells cm^−2^ determined to be ideal for producing a uniform epithelial monolayer (Figure S2b, Supporting Information).

After identifying dSIS–NB gel stiffness suitable for long‐term intestinal cell culture, cell spreading was tracked over 3 weeks using live cell imaging (Figure S3, Supporting Information). A cellular network covering more than 50% of the dSIS–NB hydrogel surface was observed by day 7, and full coverage occurred within 13 days (Figure S3a and Video S1, Supporting Information). In contrast, Caco‐2 cell aggregates formed on Matrigel covered only ≈30% of the surface after 21 days (Figure 1e; Figure S3a and Video S2, Supporting Information). The maturation of Caco‐2 cells after 21 days of culture was verified by higher expression of key intestinal epithelial genes, including *VIL‐1*, *MDR1*, *CCND1*, *ALPI*, and *SLC15A* (Figure S4, Supporting Information). *VIL‐1* and *CCND1* were particularly upregulated in cells grown on dSIS–NB hydrogels, highlighting their role in promoting functional monolayer formation and cell proliferation, respectively.

dSIS–NB hydrogels also supported faster spreading of HT29‐MTX cells, a mucus‐secreting goblet‐like cell line.^[^ ^27^ ^]^ However, unlike Caco‐2 cells that formed flattened morphology on dSIS–NB hydrogels, HT29‐MTX cells formed multicellular clusters with less spreading, reaching ≈88% confluence after 21 days (Figure 1f; Figure S3b, Supporting Information). Mixing Caco‐2 and HT29‐MTX cells yielded a similar growth pattern to Caco‐2 monoculture, achieving complete coverage of the dSIS–NB hydrogel surface within 13 days (Figure 1g; Figure S3c, Supporting Information). In contrast, co‐culture on Matrigel never reached monolayer coverage. As Matrigel was substantially softer than dSIS–NB hydrogels, we performed another control experiment using gelatin–norbornene (GelNB) hydrogels with similar stiffness to that of dSIS–NB hydrogels (i.e., *G*′ ≈ 1 or 2 kPa). Live cell imaging of Caco‐2/HT29‐MTX cells on GelNB hydrogels revealed limited cell expansion over 4 days on GelNB gels, regardless of gel stiffness (Figure S5a,b, Supporting Information), suggesting both matrix stiffness and compositions are important in promoting the proliferation of intestinal cells. Additionally, Caco‐2/HT29‐MTX cells seeded on transwell membrane achieved a confluent cell monolayer within 4 days of culture (Figure S5c, Supporting Information).

Images from live cell tracking over 21 days suggest that stiffer dSIS–NB hydrogels (*G*′ ≈ 2 kPa) support long‐term intestinal cell culture (Figure S3, Supporting Information). On dSIS–NB hydrogels, both Caco‐2 monoculture (Figure S6, Supporting Information) and Caco‐2/HT29‐MTX coculture (Figure 1h; Video S3, Supporting Information) formed a thin flat monolayer (20–25 µm), with strong CD326 (EpCAM) and uniform E‐cadherin (E‐Cad) expression. In contrast, cells formed large (600–700 µm in diameter) and thicker (120–130 µm in thickness) clusters on Matrigel (Figure 1h; Figure S6 and Video S4, Supporting Information). Additionally, more uniform zonulaoccludens‐1 (ZO‐1) expressions were found in Caco‐2 monoculture on dSIS–NB hydrogels but not in cell aggregates formed on Matrigel, while co‐culture weakened the expression of ZO‐1 (Figure 1h), suggesting a weaker tight junction than in Caco‐2 monoculture.^[^ ^28^ ^]^ The addition of HT29‐MTX cells also led to Mucin‐2 secretion on both matrices, but the accumulation of Mucin‐2 was more pronounced and wide spread on dSIS–NB hydrogels than on Matrigel (Figure 1h). TEER measurements confirmed the intestinal barrier integrity in Caco‐2 monoculture and Caco‐2/HT29‐MTX co‐culture (Figure 1i).^[^ ^29^ ^]^ Low TEER values were detected from cells cultured on Matrigel regardless of cell compositions and HT29‐MTX monoculture on dSIS–NB hydrogels, reflecting their lack of monolayer coverage (Figure 1e–g; Figure S3, Supporting Information). The incorporation of HT29‐MTX cells also caused ​​a slight reduction in TEER value (624 Ω cm^2^) as compared to Caco‐2 monoculture (746 Ω cm^2^; Figure 1i). This result is consistent with the disrupted ZO‐1 expression patterns in Caco‐2/HT29‐MTX coculture (Figure 1h). The notable differences in cell behaviors could be attributed to hydrogel stiffness and composition. The dSIS–NB hydrogel, with its adjustable mechanical properties (*G*′ ≈ 2 kPa), provided the necessary mechanical cues for cell attachment and spreading. This was in stark contrast to the performance of Matrigel, where Caco‐2 cells exhibited only limited growth and multicellular clustering, likely due to the low stiffness of Matrigel (*G*′ < 100 Pa). Scaffold stiffness is widely recognized as an important factor in regulating cell behavior, particularly for epithelial cells.^[^ ^30^ ^]^ Previous studies have shown that soft substrates can limit epithelial cell spreading and promote cluster formation rather than the formation of a continuous monolayer.^[^ ^31^, ^32^ ^]^ Zhang et al. used a tunable matrix composed of Matrigel and synthetic oligo(ethylene glycol)‐grafted polyisocyanides to show that cell morphology shifted from round hollow cysts to 2D monolayers at higher matrix stiffness.^33^ Our proteomics results also show that collagen I, collagen III, and Fibrillin made up more than 95% of dSIS and dSIS–NB (Figure S1, Supporting Information), whereas Matrigel was rich in laminin and collagen IV. The differences in biochemical compositions may also contribute to the different cell morphologies observed between dSIS–NB hydrogels and Matrigel.

### 2.2 SHM for Inverse Molding of Bioactive dSIS–NB Hydrogels with Crypt/Villus Structures

**Figure 2:**
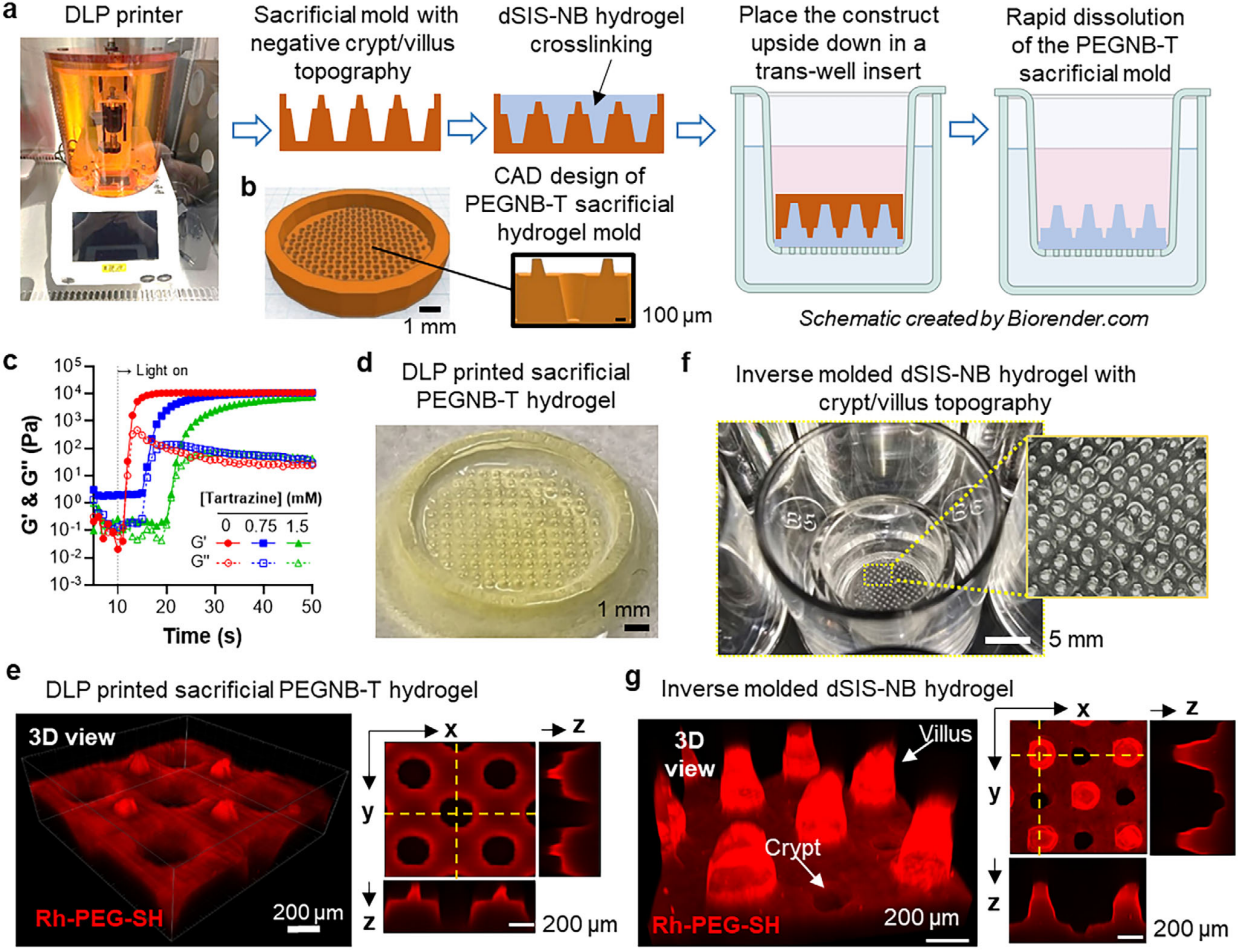
Inverse molding of bioactive dSIS–NB hydrogels with crypt/villus structure. a) Workflow of the inverse molding to generate dSIS–NB hydrogels with a positive crypt/villus topography. This process utilizes rapid and autonomous dissolution of sacrificial hydrogels in a transwell insert, allowing for the precise formation of crypt/villus structures in the dSIS–NB hydrogel. b) Design of the negative crypt/villus mold by Tinker CAD for DLP printing. c) In situ photorheometry of PEGNB‐T (4 wt%) crosslinked with PEG4SH (3 wt%) and 10 mm LAP under 405 nm light at 34 mW cm^−2^. The gelation was modulated by the addition of photoabsorber tartrazine at concentrations of 0, 0.75, and 1.5 mm, which corresponded to gel points of ≈2, ≈5, and ≈12 s, respectively, indicating tunable photopolymerization speed. d) A representative DLP‐printed sacrificial PEGNB‐T hydrogel with negative crypt/villus structures. e) 3D reconstruction of a confocal z‐stack view (left) and cross‐section view (right) of the printed PEGNB‐T hydrogel with negative crypt/villus structure. A trace of rhodamine–PEG–thiol (Rh‐PEG‐SH) was added after the DLP‐printing of sacrificial PEGNB‐T hydrogel for visualization. f) dSIS–NB crypt/villus hydrogel placed in a transwell insert following the degradation of the sacrificial PEGNB‐T hydrogel mold. g) 3D reconstruction of a confocal *z*‐stack view (left) and cross sections (right) of dSIS–NB hydrogels with positive crypt/villus structures. A trace of Rh‐PEG‐SH was added to the dSIS–NB hydrogel for visualization.

While dSIS–NB hydrogels supported the formation of monolayer intestinal epithelium within 2 weeks (Figure 1), the flat surface did not mimic the topography of intestinal tissue in vivo. One option to create crypt/villus topography is using DLP bioprinting. Although in our prior study, we have shown that dSIS–NB can be used as a bioink for 3D printing,^[^ ^22^ ^]^ its high viscosity prevents its use in DLP printing to produce the fine crypt–villus structure with a dimension between 50 and 200 µm. Therefore, an inverse molding technique was utilized where the bioactive dSIS–NB hydrogel was photocrosslinked over a SHM with negative crypt/villus structures printed by a DLP bioprinter (**Figure** **2**a). The negative crypt/villus mold was designed using Tinker CAD, with larger wells for molding villi (500 × 300 × 100 µm, for the depth, upper, and lower diameters, respectively) and small pillars for molding crypts (200 × 100 × 150 µm for the height, upper, and lower diameters, respectively) (Figure 2b). The dSIS–NB precursor solution was cured over SHM, which undergoes rapid dissolution upon contact with PBS at 37 °C (Figure 2c). PEGNB‐T was synthesized via reacting PEGNB carboxylate (PEGNB_CA_) with tyramine using standard carbodiimide chemistry (Figure S7, Supporting Information). Similar to other norbornene‐modified macromers,^[^ ^34^, ^35^ ^]^ the crosslinking of PEGNB‐T was rapid under 405 nm light, even in the presence of photoabsorber tartrazine (Figure 2d), which was added to reduce out‐of‐focus polymerization and improve the printing fidelity. The PEGNB‐T SHM was printed (Figure 2e,f; Video S5, Supporting Information) with high fidelity (90% for the negative crypts and nearly 100% for the negative villi; Figure S8a, Supporting Information).

Following DLP printing of SHM, dSIS–NB/PEG4SH/LAP solution was pipetted into the mold and crosslinked by thiol–norbornene photoclick reaction. The construct was inverted and placed in a transwell insert (Figure 2g) to allow for the rapid and autonomous dissolution of PEGNB‐T SHM. Positive crypt/villus topography on dSIS–NB hydrogel (8 mm in diameter) was revealed after the dissolution of SHM, which occurred in 2 h without any manual peeling or addition of any degradation‐facilitating reagent (Figure S9, Supporting Information). The average villus height and crypt depth were 318 and 88 µm, and the average diameters of the villus base and tip were 228 and 99 µm, respectively (Figure 2h **;** Video S6, Supporting Information). Molded dSIS–NB crypt/villus hydrogel achieved high fidelity and consistent uniformity (*p* > 0.05; Figure S8b, Supporting Information).

Compared with prior studies using gelatin methacryloyl (GelMA) and PEG–diacrylate,^[^ ^19^, ^36^ ^]^ our biofabrication method presents unique advantages, including the use of highly bioactive dSIS matrices and high‐fidelity DLP‐printing of autonomous PEGNB‐T SHM. Elomaa et al. used DLP printing to create villus structures from dSIS–methacrylate (dSIS‐MA).^[^ ^17^ ^]^ Unfortunately, the high viscosity of dSIS–MA resulted in low fidelity (≈58%), high swelling (30–40%) post printing, and a lack of crypts. Rudolph et al. employed a similar inverse‐molding technique to produce silk‐based scaffolds featuring crypt/villus structures.^[^ ^20^ ^]^ However, the multistep process involved: 1) DLP‐printing of a polymer resin, 2) casting of PDMS negative mold, 3) coating and curing of a silk film on the PDMS mold, 4) curing of silk‐spongy scaffold, 5) manual peeling of the scaffold, and 6) coating of collagen to provide cell adhesion sites. In contrast, our PEGNB‐T SHM was DLP‐printed in a single step, followed by rapid gelation of dSIS–NB gels over the SHM without the need for any coating. The removal of SHM occurred autonomously through rapid hydrolysis and without user intervention. This simple process created bioactive dSIS–NB hydrogels with physiologically relevant stiffness (*G*′ ≈ 2 kPa) and intricate crypt/villus topography.

### 2.3 Intestinal Cell Behaviors on Bioactive dSIS–NB Hydrogels with Crypt/Villus Structure

**Figure 3:**
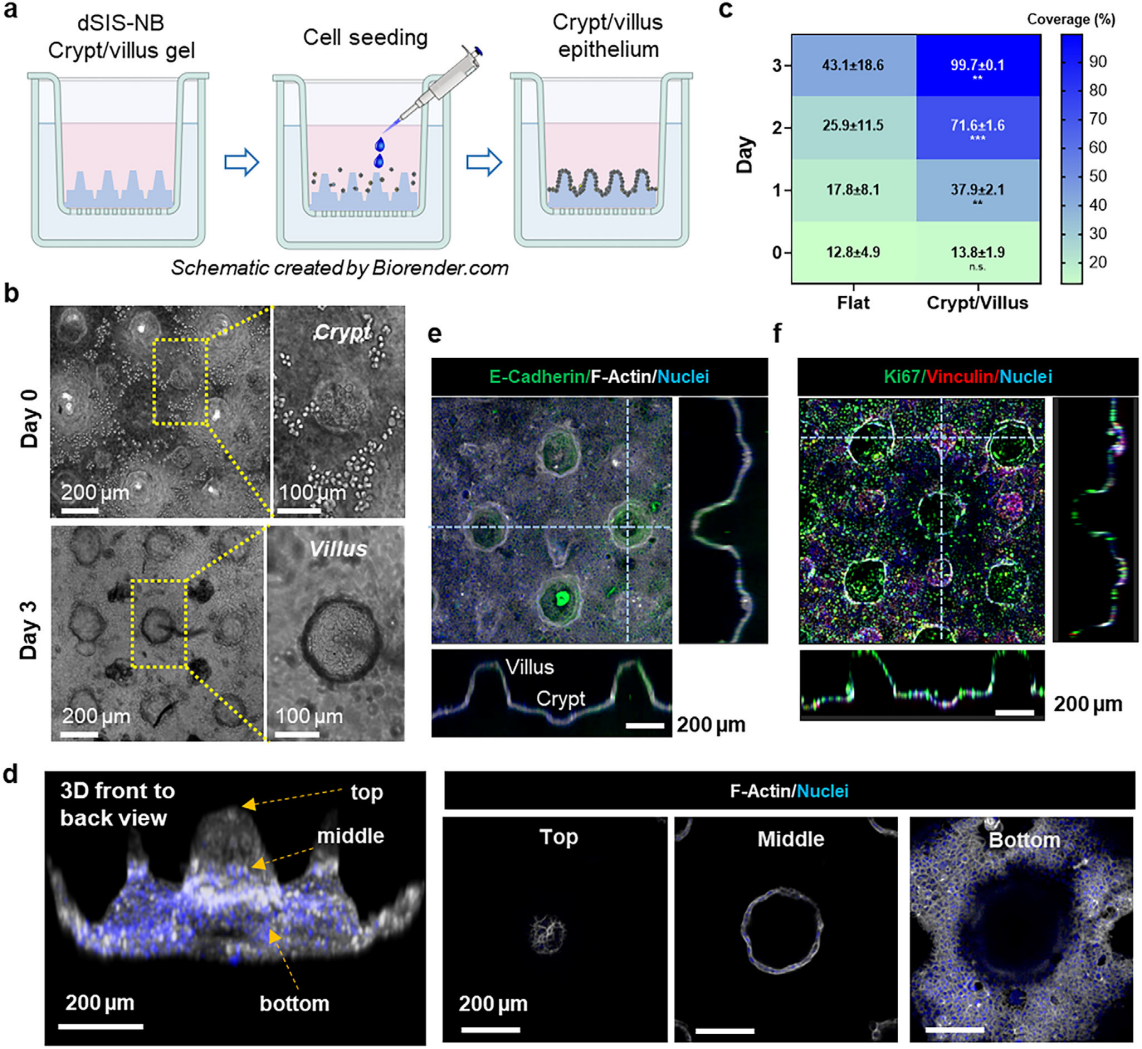
Culture of intestinal cells on bioactive dSIS–NB hydrogels with crypt/villus structure. a) Schematic of cell seeding and monolayer formation on the inverse‐molded dSIS–NB hydrogel with positive crypt/villus structure. b) Bright‐field images of Caco‐2/HT29‐MTX cells distributed on the scaffold surface after seeding (i.e., day 0) and day 3. Cell clustering in a crypt on day 0 and a full coverage of a villus on day 3 were highlighted. c) Heat map showing the average local cell coverage on 2D flat and 3D crypt/villus dSIS–NB hydrogels. Coverage was assessed from four random regions of each hydrogel, with three independent replicates per condition. Data are presented as mean ± SEM. Cell coverage reached nearly 100% on dSIS–NB gel with crypt/villus topography on day 3, outperforming flat hydrogels. n.s.: Nonsignificant; ** and *** represent *p* < 0.01 and *p* < 0.001, respectively, by multiple paired *t*‐tests at the same time point. d) 3D confocal front‐to‐back view of an intestinal epithelium (stained with F‐actin (white) and nuclei (blue) formed over the dSIS–NB hydrogels with crypt/villus topography on day 3 of culture. Zoom‐in images show the cell coverage on the top, middle, and bottom sections of a villus. e) Representative immunostaining images of E‐cadherin (green) and F‐actin (white) of day 3 to assess cell–cell interaction and cytoskeleton structure, respectively. Nuclei were counter‐stained with 4′,6‐diamidino‐2‐phenylindole ((DAPI (blue)). Strong E‐cadherin expression was observed on the villi. f) Representative immunostaining images of Ki67 (green) and vinculin (red) on day 3 to assess cell proliferation and focal adhesion complex formation, respectively. Ki67‐positive cells were evenly distributed along crypt/villus axis, suggesting active cell division, while vinculin, marking focal adhesion sites, was concentrated in the crypts, reflecting stronger cell–extracellular matrix interactions in these areas. Nuclei were counter‐stained with DAPI (blue).

The formation of intestinal cell monolayer on dSIS–NB hydrogels with crypt/villus topographies was evaluated using intestinal epithelial cells Caco‐2 and mucus‐secreting HT29‐MTX cells (**Figure** **3**a). After seeding, cells clustered predominantly within the crypts and surrounding the bases of the villi, and very few cells were found on the tips of villi (Figure 3b, day 0). Over time, the cells proliferated to cover the remaining area and eventually formed a complete epithelial monolayer within 3 days (Figure 3b, day 3; Video S7, Supporting Information). This was significantly faster than the flat 2D culture counterpart, which took 13 days to reach complete surface coverage (Figure 1). While cell coverage on both flat dSIS–NB gel surface and crypt/villus structure was about 12–13% of the gel surfaces following initial cell seeding (Figure 3c, day 0), cells seeded on 2D flat surfaces clustered randomly, with a large portion of the area void of cells, prolonging the time it took to reach full coverage (Video S1, Supporting Information). After 1 day, cells on the flat surface had just begun spreading in a few regions, reaching about 18% confluence, while cells on crypt/villus scaffolds had already started spreading at the base of villi, achieving about ≈38% confluence (Figure 3c, day 1). Monolayer formation over the entire crypt/villus structure was verified through immunofluorescence staining of F‐actin (Figure 3d; Figure S10 and Videos S8 and S9, Supporting Information). On the first day, cells efficiently clustered together in the crypt while cells on the base of villi began to spread. By the second day, the crypt and villus bases were almost completely covered while no cells were found on the villus projections. Impressively, a complete cell monolayer covering the entire crypt/villus structure was observed by the third day. In contrast, cells on the flat surface still exhibited high variation in local cell coverage, with some areas full of cells while others showed very low coverage (Figure 3c; Figure S11a,b, Supporting Information). Additional immunostaining of E‐cadherin (Figure 3e), as well as Ki67 and vinculin (Figure 3f; Figure S11c,d, Supporting Information) revealed that the cells were highly proliferative and formed strong focal adhesion complexes throughout the 3D crypt/villus surface than on 2D flat surface (Figure S11, Supporting Information). In a previous work, Castaño et al.^[^ ^37^ ^]^ used a photomask to generate villi‐like pillars on much stiffer poly(ethylene glycol) diacrylate (PEGDA) hydrogels (>10 kPa). While Caco‐2 monolayer was achieved in ≈50 h, the PEGDA hydrogel did not contain crypts, and a secondary protein conjugation step was needed to allow cell attachment on the otherwise inert PEGDA gel surface. In contrast, our dSIS–NB hydrogels were inherently cell adhesive and the inverse‐molding process created both crypts and villi on gels with physiological relevant stiffness (≈2 kPa). Evenly distributed crypts may act as cell “niches” that promote early cell aggregation and efficient cell‐to‐cell interaction, leading to faster spreading. All together, these results suggest that the topographical features of the crypt/villus structure not only promote more uniform cell seeding but also enhance initial cell adhesion and even spreading, resulting in more efficient coverage compared to flat surfaces.

### 2.4 Epithelial Polarization and Tight Junction Formation

**Figure 4:**
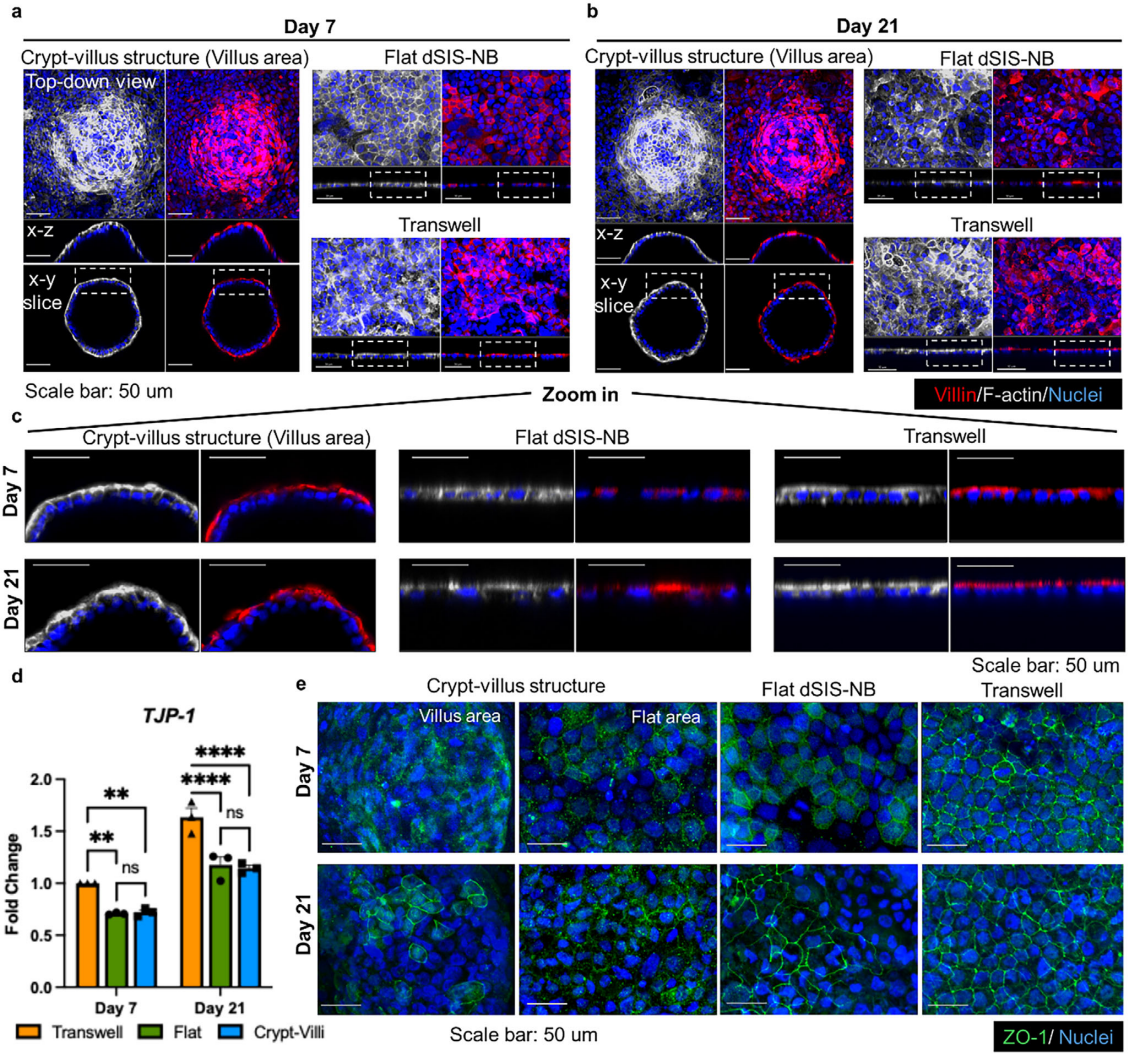
Evaluation of epithelial polarization and tight junction on different scaffolds. a,b) Representative confocal images of Caco‐2/HT29‐MTX monolayers cultured on crypt–villus dSIS–NB hydrogels, flat dSIS–NB hydrogels, and conventional transwell membrane at a) day 7 and b) day 21. Cells were stained for Villin (red) to visualize the brush border and microvilli formation. F‐actin (white) was stained to assess cytoskeletal organization. Nuclei were counterstained with DAPI (blue). c) Higher magnification of the dashed box regions. The orientation of the monolayers shows the apical side facing up and the basal side facing down. d) messenger RNA (mRNA) expression of TJP‐1, the gene encoding tight junction protein ZO‐1. Data are presented as mean ± SEM (*n* = 3, ^*^ *p* < 0.05, ^**^ *p* < 0.01, ^***^ *p* < 0.001, and ^****^ *p* < 0.0001 by two‐way ANOVA multiple comparisons Tukey's posthoc test. e) Immunofluorescence staining of ZO‐1, a tight junction protein.

To further assess epithelial polarization, we performed immunofluorescence staining and imaging of Villin, a well‐established marker of brush border and microvilli formation (**Figure** **4**a–c). Caco‐2/HT29‐MTX monolayers were cultured on crypt–villus dSIS–NB hydrogels, flat dSIS–NB hydrogels, and conventional transwell membranes. On day 7, cells on crypt–villus hydrogels and transwell membranes displayed a polarized phenotype, with Villin detected on the epical surface and absent in the basolateral side. Similarly, F‐actin was enriched at the apical membrane, while also outlining intercellular junctions along the apical–basolateral border. In contrast, cells grown on flat dSIS–NB hydrogels on day 7 showed less distinct polarization, as F‐actin was distributed more evenly between apical and basolateral surfaces (Figure 4a,c). This could be attributed to the fact that cells on crypt/villus surface and transwell membrane reached confluence in 3–4 days (Figure 3), but it took 13 days on flat dSIS–NB hydrogel (Figure 1). By day 21, all three conditions exhibited well‐polarized epithelial monolayers, with Villin and F‐actin signals being concentrated at the apical surface (Figure 4b,c). These results indicate that the crypt–villus topography accelerates polarization and brush border maturation compared to flat hydrogel surfaces, supporting its role in promoting more physiologically relevant epithelial organization.

The extent of tight junction formation was further evaluated at both mRNA (“TJP‐1”) and protein (ZO‐1) levels. Quantitative reverse transcription‐polymerase chain reaction (RT‐PCR) revealed that “TJP‐1” expression was consistently higher in cells cultured on transwell than on dSIS–NB hydrogel (Figure 4d). Immunostaining of ZO‐1 showed more intense and continuous tight junctions along each cell border in transwells, whereas cells on flat and crypt–villus dSIS–NB hydrogels exhibited weaker and less continuous staining at both time points (Figure 4e). These results indicate that transwell cultures with excessively high expression of tight junction proteins may hinder molecular transport across the intestinal epithelium. Although “TJP‐1” transcript levels were comparable between flat and crypt–villus dSIS–NB hydrogels, ZO‐1 protein distribution revealed important structural differences. The lower and more spatially heterogeneous ZO‐1 signal on crypt–villus hydrogel may reflect localized regulation of junctional complexes in response to curvature and microtopography. Together, these results highlight that scaffold geometry not only influences the degree of tight junction formation but also their spatial organization.

Taken all together, these results highlight the distinct roles of scaffold topography and composition in guiding epithelial polarization. The crypt–villus hydrogel accelerated polarization and brush border formation, as shown by earlier apical localization of Villin and F‐actin compared to flat hydrogels. However, when examining tight junction formation, both hydrogel‐based models showed lower TJP‐1/ZO‐1 expression compared to transwells, which are known to generate overly tight barriers not representative of the human intestine. This suggests that the dSIS–NB hydrogel provides a more physiologically relevant environment for epithelial organization and barrier function, balancing polarization and tight junction formation in a way that better mimics the in vivo intestinal epithelium. The combination of early formation of polarized epithelium from the crypt–villus structure and more moderate tight junction expression resulting in a model that aligns more closely with physiological conditions.

### 2.5 Assessment of Barrier/Absorption Function

**Figure 5:**
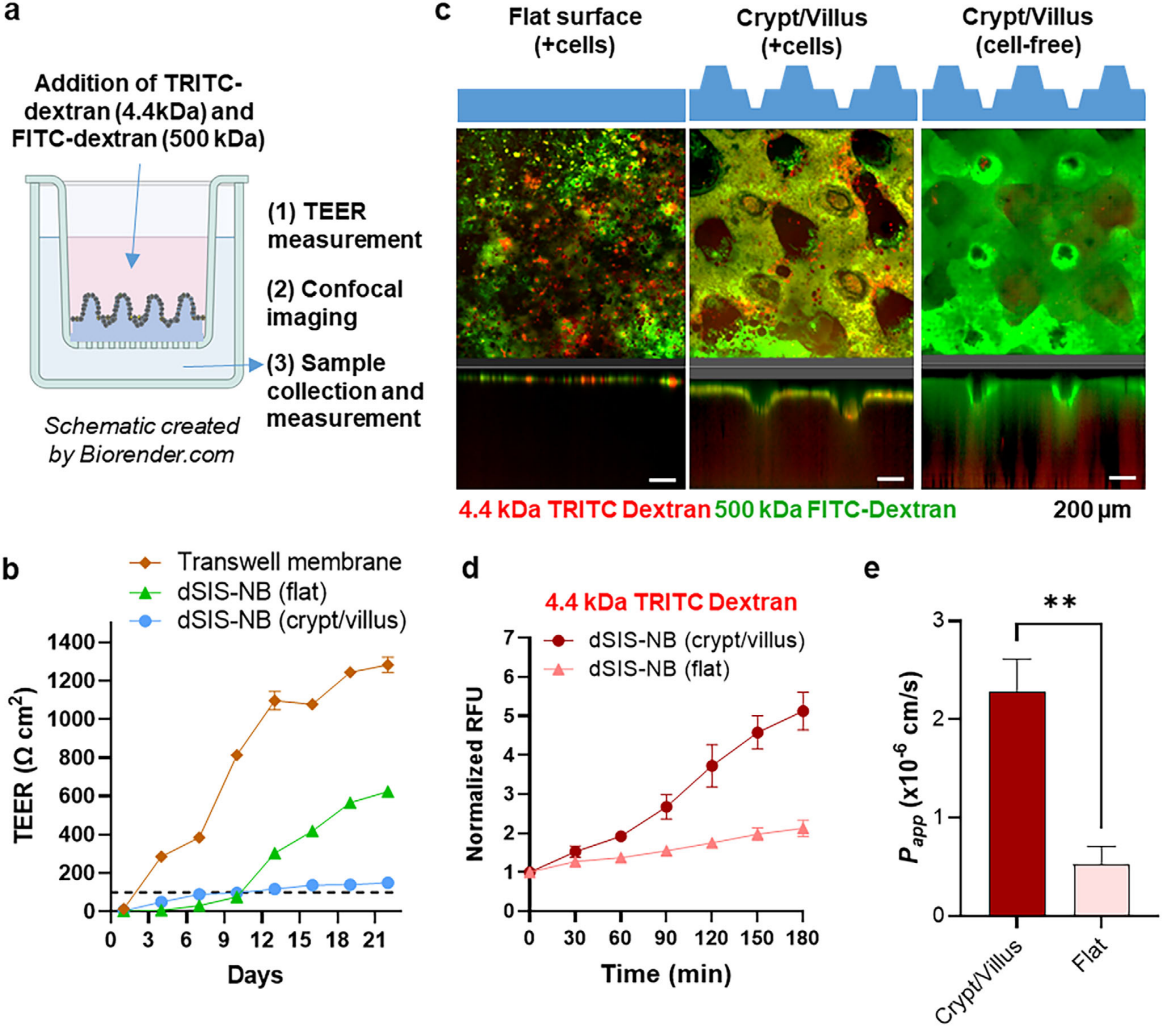
Functional characteristics of 2D flat and 3D crypt/villus models. a) Schematic of crypt/villus barrier integrity and permeability assays. b) TEER measurements of the intestinal epithelial barrier on conventional transwell, 2D flat, and 3D crypt/villus models. Data are presented as mean ± SEM (*n* = 4). The dashed line indicates a physiological TEER value (in vivo) obtained from Srinivasan et al.^[^ ^29^ ^]^ c–e) Permeability studies using dextran permeation from the apical to basolateral side for 2D flat and 3D crypt/villus models: c) confocal images of 4.4k‐TRITC‐dextran (TD) (red) and 500k‐FD (green) permeation in dSIS–NB hydrogels with flat (cell‐laden, left), 3D crypt/villus structure (cell‐laden, middle), and cell‐free hydrogel (control, right); d) quantification of fluorescence intensity for 4.4k‐TD permeation from the apical to basolateral side of 2D flat and 3D crypt/villus dSIS–NB models. Normalized relative fluorescence units (RFU) values were determined by the RFU(*t_n_*)/RFU(*t*_0_) ratio (*n* = 3); and e) calculated apparent permeability coefficients (*P*_app_) from 4.4k‐TD permeation assays. Data are presented as mean ± SEM (*n* = 3, **p < 0.01 by a two‐tailed Student's *t*‐test). The 3D crypt/villus model exhibited a significantly higher *P*_app_ value compared to the 2D flat model, indicating enhanced permeability function in the 3D model.

To assess the long‐term epithelium viability and stability in our 3D dSIS–NB crypt/villus model, the structure was stained with EpCAM, E‐cadherin, and F‐actin after 21 days of in vitro culture (Figure S12, Supporting Information). The staining results revealed that all crypts and villi remained intact after 21 days, with no visible defects in the monolayer. Longitudinal monitoring of Caco‐2/HT29‐MTX co‐culture revealed that the rigid transwell inserts yielded a rapid rise of TEER values, increasing significantly from 287 Ω cm^2^ on day 4 to 1284 Ω cm^2^ on day 22 (**Figure** **5**b). Meanwhile, cells cultured on flat dSIS–NB hydrogels showed minimal TEER increases (under 50 Ω cm^2^) for the first 10 days, followed by a sharp increase afterward (76 Ω cm^2^ on day 10 to 304 Ω cm^2^ on day 13) when the cells reached full surface confluence (Figure 1g). TEER values were higher for 3D crypt/villus gel than on the flat counterpart during the first week, with values rising from 6 Ω cm^2^ on day 1 to 50 Ω cm^2^ by day 4, reflecting the rapid epithelialization over the crypt/villus architecture (Figure 3). Note that although the cells reached a complete monolayer after 3 days of culture on the crypt/villus model, they might not be fully differentiated and polarized. The TEER value continued to increase over time and reached a more stable value on day 7, which is consistent with the polarized phenotype of cell culture on crypt/villus structure (Figure 4). After 1 week, the 3D crypt/villus model presented a limited increase, exhibiting values within a range of 90–150 Ω cm^2^ from day 7 to day 22. These values were comparable to those obtained from in vivo intestinal tissues (40–100 Ω cm^2^).^[^ ^29^, ^38^, ^39^ ^]^ As the total surface of the crypt/villus hydrogel was bigger than flat surface, we accounted for the total villi and crypt numbers and the surface area in each sample. In total, 147 villi and 145 crypts were formed in a single scaffold. The effective surface area of crypt/villus hydrogel is 0.74 cm^2^, only 23% higher than the flat hydrogel or transwell membrane (0.60 cm^2^). When normalized to the increased surface area, the TEER value of 3D crypt/villus model corresponded to values from 112 to 185 Ω cm^2^ over day 7 to day 22. These normalized values ​​were still much closer to the physiological range in native intestinal tissue than in the flat hydrogel and transwell models.

Permeability studies were conducted using fluorescently labeled dextran: 4.4 kDa tetramethylrhodamine isothiocyanate‐dextran (4.4k‐TD) and 500 kDa fluorescein isothiocyanate‐dextran (500k‐FD). Thick dSIS–NB hydrogels (thickness ≈ 1.15 mm) with flat and crypt/villus topographies were used for visualizing dextran distribution across the intestinal epithelial layer by confocal microscopy (Figure 5c). On flat hydrogels with cell monolayer coverage, most of the dextran, regardless of molecular size (4.4k‐TD or 500k‐FD), was retained at the apical surface of the intestinal cell layer, with very weak fluorescence signal observed in the hydrogel layer (Figure 5c, flat surface + cells). However, cell‐laden dSIS–NB crypt/villus hydrogel provided selective barrier function for 500k‐FD but allowed the gradual passage of 4.4k‐TD, as demonstrated by higher red fluorescence signals in the hydrogel layer (Figure 5c, crypt/villus + cells). As expected, cell‐free 3D crypt/villus dSIS–NB hydrogel did not provide barrier function, as both 4.4k‐TD and 500k‐FD could be observed in the hydrogel layer (Figure 5c, crypt/villus cell free). These results indicate that intestinal epithelium formed on both flat and crypt/villus hydrogels restricted the passage of larger molecules (i.e., 500k‐FD), consistent with the restrictive nature of tight junctions for high‐molecular‐weight molecules. Interestingly, small molecules (i.e., 4.4k‐TD) were gradually transported across the crypt/villus epithelium, a phenomenon akin to the selective permeability observed in native intestinal tissue.

Next, transport of 4.4k‐TD from the apical to the basolateral side was quantified in both models. Consistent with the confocal images, 4.4k‐TD showed a progressive increase in transport across the 3D crypt/villus model over time, while transport in the flat model remained minimal (Figure 5d). Furthermore, the apparent permeability coefficient (*P*_app_) for 3D crypt/villus model was nearly fivefold higher than that in the flat counterpart (2.3 × 10^−6 ^vs 0.5 × 10^−6^ cm s^−1^, respectively, Figure 5e). After normalization for the increased effective surface area, the *P*_app_ of the crypt/villus model remained higher than that of flat hydrogel (1.9 × 10^−6^ vs 0.5 × 10^−6^ cm s^−1^), further highlighting the impact of tissue architecture on absorption function.^[^ ^37^, ^40^ ^]^

In addition to increasing surface area, the crypt/villus topography also alters epithelial behavior in other ways. The expression patterns of ZO‐1 in cells over crypt/villus hydrogel were discontinuous and uneven, suggesting weakened intracellular junctions. In contrast, ZO‐1 patterns in flat dSIS–NB gels were more organized but still uneven. Finally, ZO‐1 expressions in cells on transwell membrane were highly organized, suggesting very tight cell–cell junctions. These results explain the higher *P*_app_ and lower TEER values of the crypt/villus model compared to conventional models. The combination of an increased surface area and looser barrier facilitates nutrient and drug absorption while maintaining selective permeability. Noted that, although our 3D crypt/villus model achieved full epithelial confluence within 3 days (Figure 3), we performed functional permeability assays on day 21 to ensure a fair comparison with the flat hydrogel control, which required up to 13 days to reach full confluence. Moreover, testing on day 21 also demonstrated the long‐term stability and sustained barrier integrity of the epithelial monolayer maintained on the dSIS–NB crypt/villus scaffold.

### 2.6 Crypt/Villus Structure Used as a Model for Gastrointestinal Toxicity

**Figure 6:**
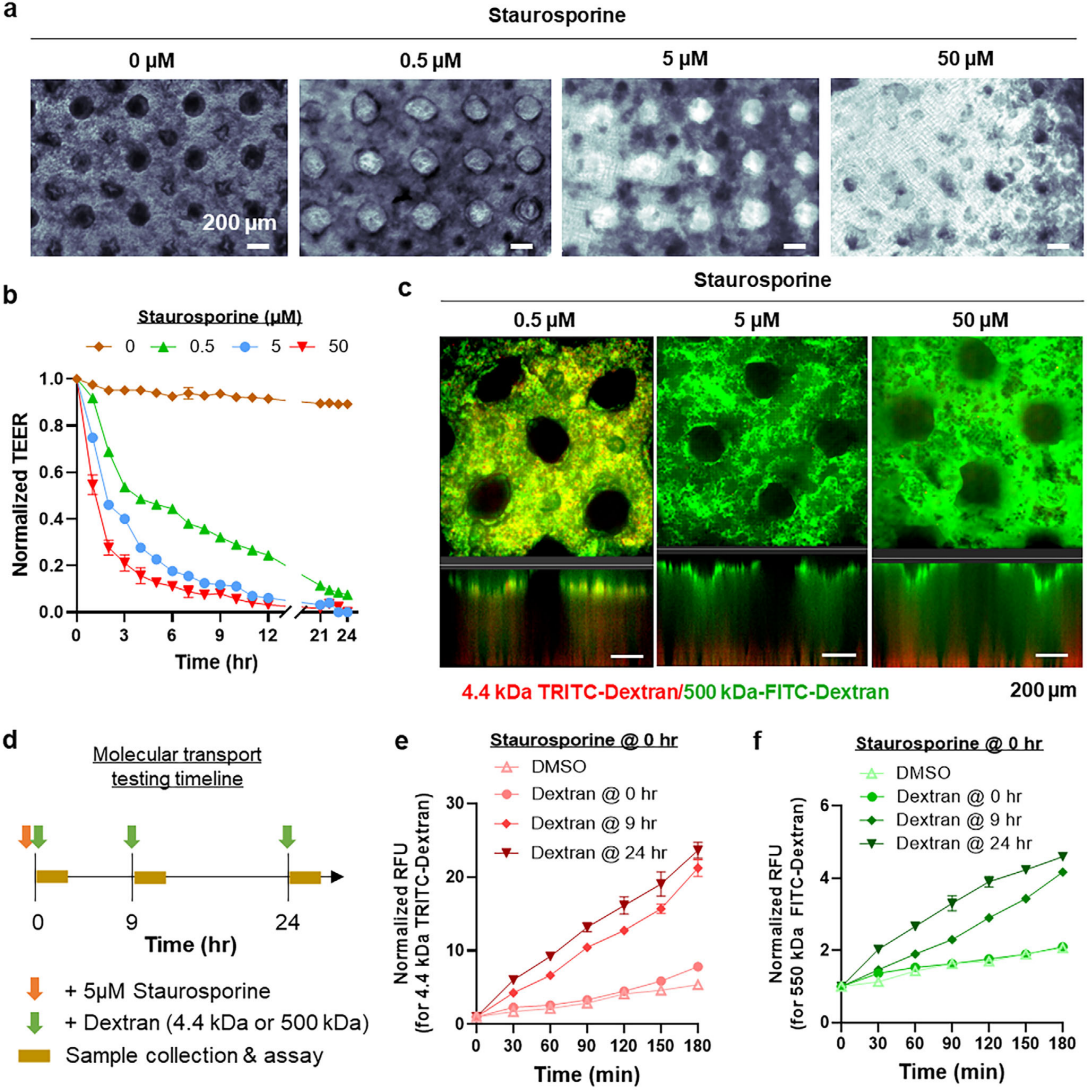
Drug‐induced loss of crypt/villus barrier integrity. a) Bright‐field images of the cell‐laden crypt/villus layers under toxic stress conditions with different concentrations of staurosporine (after 24 h of exposure). b) Epithelial barrier disruption with different staurosporine concentration evaluated by TEER values over time for 24 h. Normalized TEER values were determined by the TEER(*t_n_*)/TEER(*t*_0_) ratio (*n* = 3). c) Confocal images present dextran leakage of 3D crypt/villus model after 24 h of staurosporine exposure using 4.4k‐TD and 500k‐FD. d) Timeline of the toxicity and transport assay. staurosporine was added at the start of the experiment, while dextran was added at the time indicated post‐staurosporine addition (i.e., 0, 9, and 24 h). e,f) Effect of staurosporine (5 µm) on permeability of e) 4.4k‐TD and f) 500k‐FD in dSIS–NB hydrogel with crypt/villus structure. DMSO was used as a control to establish the baselines of molecular transport in the absence of drug treatment.

Having demonstrated the successful creation of a “functional” 3D crypt/villus intestine model, we explored its potential to model barrier “dysfunction,” such as inflammatory bowel disease (IBD), indigestion, irritable bowel syndrome, and celiac disease (CD).^[^ ^41^, ^42^, ^43^ ^]^ The intestinal epithelium formed on the dSIS–NB crypt/villus hydrogel was challenged with staurosporine, a broad‐spectrum kinase inhibitor widely used to induce cellular apoptosis.^[^ ^44^, ^45^ ^]^ A clear dose‐dependent disruption of the intestinal epithelium was observed after 24 h of staurosporine treatment (**Figure** **6**a). Staurosporine‐induced loss of intestinal epithelium integrity was verified via rapid and dose‐dependent decreases of TEER values (Figure 6b). High concentrations (5 and 50 µm) of staurosporine treatment also clearly increased the transport of 500k‐FD (Figure 6c), a phenomenon not observed under drug‐free conditions (Figure 5c), indicating a severe breakdown of tight junction integrity. Molecular transport studies were conducted using 4.4k‐TD and 500k‐FD added to the crypt/villus model at different time points post‐staurosporine exposure (i.e., 0, 9, and 24 h; Figure 6d). When 4.4k‐TD and 500k‐FD were added at the same time as staurosporine treatment (i.e., dextran at 0 h), no drastic changes were observed in molecular transport compared with the dimethyl sulfoxide (DMSO) control, suggesting that the rapid reduction of TEER values (Figure 6b) preceded the changes in molecular transport. When 4.4k‐TD and 500k‐FD were added 9 or 24 h post‐staurosporine treatment (i.e., dextran at 9 and 24 h), significant increases in both 4.4k‐TD and 500k‐FD transport across the crypt/villus intestinal layer were detected (Figure 6e,f). A clear increase in *P*_app_ was noted for both dextran sizes over time after staurosporine exposure (Figure S13, Supporting Information), indicating that the drug disrupted the epithelial barrier, causing higher permeability. Our data indicated that the TEER measurement is more sensitive to early epithelial barrier damage than the fluorescent leakage assay. The immediate decline in TEER values upon exposure to staurosporine highlights the sensitivity of TEER as an early indicator of tight junction dysfunction. In the context of drug testing for gastrointestinal toxicity, TEER can serve as a rapid, noninvasive measurement to identify compounds that compromise barrier function. In contrast, the fluorescent leakage assay provided valuable insights into molecular permeability, though its sensitivity lagged behind TEER response. No significant fluorescent leakage was observed in the first 2 h, suggesting that while tight junctions were compromised, the barrier was still able to restrict molecular diffusion. However, as barrier damage progressed, increased leakage of small‐molecular‐weight probes was observed after 3 h, with clear permeability across the epithelial layer by the 9 and 24 h time points. The delayed response of the fluorescent leakage assay suggests that molecular permeability assays may miss early signs of barrier disruption, particularly when the damage primarily affects tight junctions without immediately compromising the entire epithelial layer. However, at later stages, when the barrier becomes more permeable, fluorescent leakage assays provide a complementary approach to TEER by identifying changes in molecular permeability that TEER cannot detect. The sensitivity of TEER to early‐stage barrier disruption and the molecular insights provided by fluorescent leakage assays make this approach highly effective for evaluating drug‐induced epithelial damage. This finding provides valuable insights into the utility of different assays for assessing drug effects on epithelial integrity, where TEER can serve as a rapid, noninvasive measurement to identify compounds that compromise barrier function, but the results must be confirmed with actual molecular transport studies.

### 2.7 “Pizza” Hydrogels to Model Intestinal Disease

**Figure 7:**
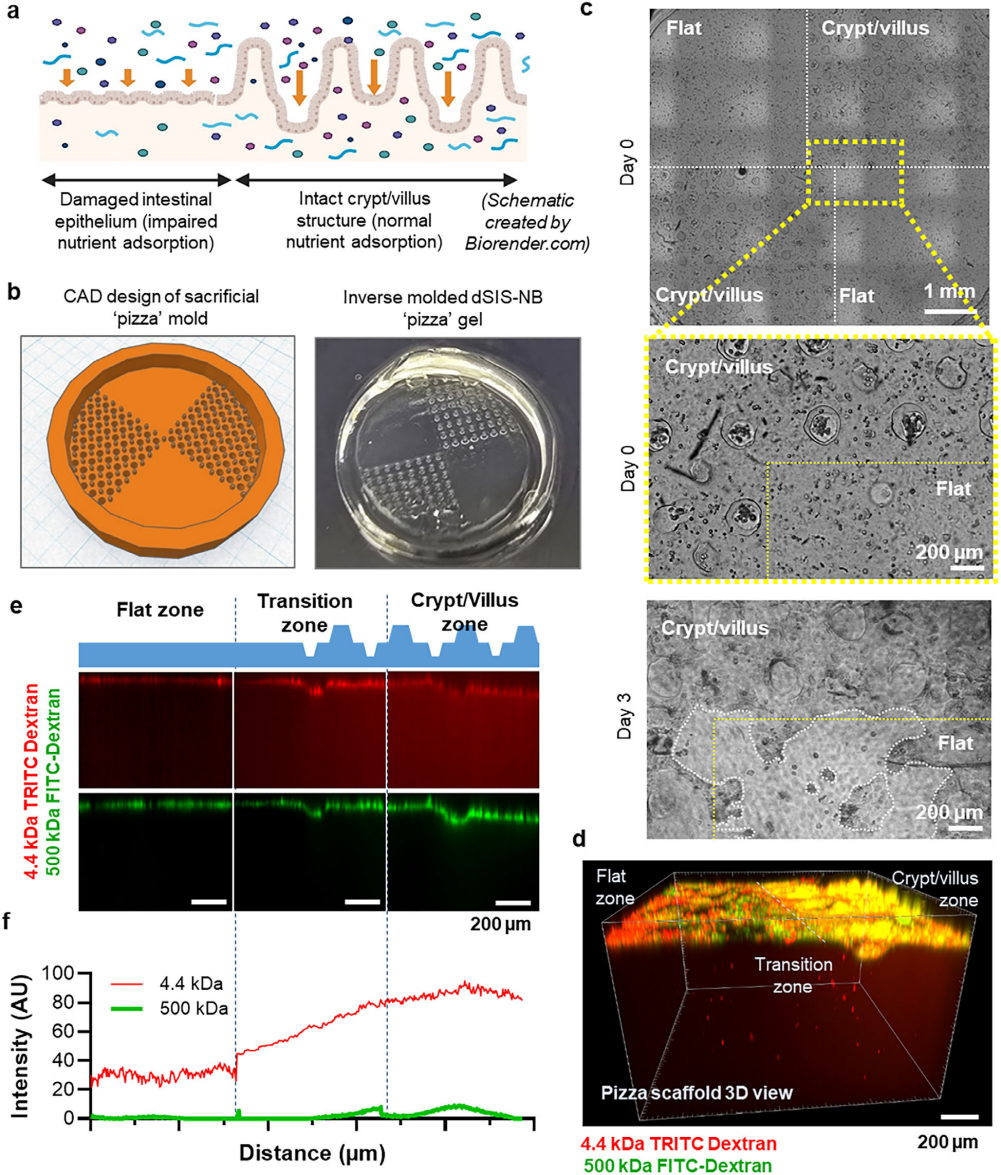
“Pizza’‐like dSIS–NB hydrogel to model intestinal disease. a) Schematic of molecular transport in celiac disease and healthy crypt/villus epithelium. b) Design of the negative pizza mold (left) by Tinker CAD and molded dSIS–NB “pizza” hydrogel (right). c) Bright‐field images of the intestinal cells culture on the “pizza” hydrogels. The upper two images indicate similar degree of cell spreading on both flat and crypt/villus structures after seeding (day 0). The bottom image shows the difference in cell coverage on day 3 between flat and crypt/villus topographies. White dashed lines mark the cell boundary while the thin yellow dashed lines mark the flat and crypt/villus topography boundaries. d–f) Permeability assay using the dSIS–NB “pizza” hydrogel with flat, transition, and crypt/villus topography: d) representative 3D overall view for dextran transport across the flat and crypt/villus epithelium on the pizza model; e) representative cross‐sectional view (*x*–*z*) of the pizza hydrogel model with dextran transport through the intestinal epithelial cell layer into the hydrogel; and f) quantification of 4.4k‐TD and 500k‐FD transport along the distance from flat to transition and to crypt/villus zones. The *y*‐axes represent the signal intensity and the *x*‐axes represent the distance corresponding with panel (e). To ensure an accurate comparison of fluorescence intensity, the whole hydrogel channel was selected.

Celiac disease is a chronic autoimmune disorder of the small intestine triggered by the ingestion of gluten in genetically predisposed individuals.^[^ ^46^ ^]^ Intestinal villous atrophy, crypt hyperplasia, and a flat intestinal surface were notable in CD,^[^ ^47^ ^]^ causing severely hindered nutrient absorption (**Figure** **7**a).^[^ ^48^ ^]^ In vitro CD models are limited in their capacity to mimic the complexity of the disease, particularly the structural changes from healthy villi to flattened mucosa. This work presents a proof‐of‐concept “pizza”‐like dSIS–NB hydrogels with features of both villus‐rich architectures of the healthy small intestine and the progressively flattened surface of celiac disease (Figure 7b), ensuring consistent experimental conditions (e.g., cell density) and allowing side‐by‐side comparisons of molecular transport. The “pizza” hydrogel was fabricated using DLP printing of sacrificial PEGNB‐T hydrogel with both flat and negative crypt/villus topographies (Figure 7b). After the crosslinking of dSIS–NB hydrogels and the dissolution of PEGNB‐T sacrificial hydrogels, Caco‐2/HT29‐MTX cells were seeded on the “pizza” dSIS–NB hydrogels. Similar to that observed in separate hydrogel cultures (Figures 1 and 3), on the “pizza” hydrogel, regions with intact crypt/villus structures were fully covered by Caco‐2/HT29‐MTX cells as early as day 3, whereas the flat regions exhibited delayed cell spreading (Figure 7c). A molecular transport study using 4.4k‐TD and 500k‐FD across the “pizza” hydrogel further revealed the clearer differences in permeability patterns across gel with different topographies (Figure 7d–f). The “pizza” hydrogel surface was separated into flat, transition, and crypt/villus zones, representing celiac disease, transition, and healthy intestinal epithelium, respectively. Similar to the transport study results shown in Figure 5, the flat zone restricted the transport of both 4.4k‐TD and 500k‐FD, whereas the crypt/villus zone permitted the passage of 4.4k‐TD and a limited amount of 500k‐FD. This observation was consistent with the notion that the healthy crypt/villus architecture promotes efficient mass transfer, while a flattened epithelium hampers normal nutrient absorption, a signature characteristic of celiac disease in vivo.

As for the benefits of the “pizza‐like” hydrogel, we believe that 1) it will allow one to observe cell proliferation in different zones without having to fabricate different scaffolds and ensures consistency in the same experimental environment, 2) it can eliminate sample‐to‐sample variability while allowing for simultaneous, side‐by‐side comparisons of molecular transport, and 3) it can capture the “patchy” appearance of celiac disease to reflect the spatial variation of villus atrophy. The dual‐region scaffold that mimics healthy (with crypt–villi) and diseased (flat) intestinal tissue in a single construct offers a simple yet effective within‐sample comparison. Our engineered dSIS–NB crypt/villus hydrogels could also be leveraged to study immune‐mediated damage to the crypt/villus architecture. To our knowledge, engineered matrices that can recapitulate the spatiotemporal variation of intestinal diseases have not been reported in the literature.

## 3 Outlook

Creating a functional small intestine model is challenging owing to the complex topography of the crypt/villus structure. This work presents a novel and robust biofabrication platform to recapitulate the 3D crypt/villus architecture of the small intestine. Specifically, we leveraged the DLP‐printed dissolvable PEGNB‐T SHM to mold cell‐responsive dSIS–NB hydrogel with the delicate crypt/villus structures. From early cell adhesion to complete monolayer formation, dSIS–NB hydrogels with crypt/villus topography outperform Matrigel. The only special instrument required for creating the crypt/villus topography is a DLP printer, which has become widely accessible in recent years. Both norbornene‐functionalized macromers, PEGNB‐T and dSIS–NB, could be synthesized in a standard fume hood, making this platform adaptable for molding matrices for in vitro modeling of epithelial diseases. Looking ahead, this platform opens new avenues for investigating the impact of various diseases on intestinal barrier physiology and function, such as IBD, where the crypt/villus structure is often disrupted. Similarly, the model could be used to investigate pathogen invasion and host defense mechanisms in gastrointestinal infections by introducing relevant microbial or viral agents to the apical surface. Additionally, the model may be adapted to study early events in colorectal cancer progression by incorporating genetically modified or patient‐derived cells into the crypt regions. Moreover, as dSIS–NB hydrogels are cytocompatible for in situ cell encapsulation,^[^ ^22^ ^]^ other stromal cells, including endothelial cells, fibroblasts, and immune cells, can be incorporated within the dSIS–NB hydrogels for modeling complex cellular dynamics. Another future direction is the adaptation of the current transwell platform to enable the application of flow or peristaltic forces, thereby creating a microphysiological system that more closely mimics the in vivo intestinal microenvironment. In sum, the simplified and highly adaptable fabrication workflow holds significant promise for advancing gastrointestinal tissue engineering and drug discovery.

## 4 Experimental Section

### Decellularization of SIS

dSIS–NB was synthesized according to the established protocols.^[^ ^22^ ^]^ Briefly, fresh bovine small intestine was collected from local grocery stores and stored at −20 °C for no longer than 1 month. 1 kg of intestine was washed carefully with tap water, then cut into 10 cm in length, and the mesenteric tissues were manually removed. The intestine segments were inverted and scrubbed to remove the mucosal epithelium and lamina propria. The segments were flipped back and the tunica serosa and tunica muscularis externa were removed. Prior to decellularization, the cleaned SIS tissues were rinsed two times with PBS, followed by stirring in 250 mL PBS containing 1% sodium dodecyl sulfate (SDS) and 2% penicillin–streptomycin for 4 days, with daily buffer change. Next, the tissues were treated with 1% triton‐X100 containing 0.05 mg mL^−1^ gentamycin for 24 h before vigorous washing in autoclaved deionized water for 7 days (change every 8–12 h). The dSIS obtained was lyophilized for 72 h under −50 °C, 20 Pa, and stored at −80 °C for use within 12 months.

### Synthesis of dSIS–NB

150 mg of desiccated dSIS was cut into small pieces using sterilized scissors and then digested in 75 mL of acidic solution (pH 2, adjusted by adding 0.01 n HCl) containing 150 units of pepsin per mg of dSIS at room temperature (RT, 20–22 °C) for 7 days until fully dissolved. Norbornene conjugation was achieved by adding 135 mg of CA to the acidic dSIS solution supplemented with ≈255 µL of triethylamine (TEA) with magnetic stirring (650 rpm) at RT for 6 h. The dSIS–NB solution was passed through a 40 µm cell strainer to remove debris, followed by dialysis (12 kDa molecular weight cutoff) against precooling autoclaved deionized (DI) water at 4 °C for 3 days, with frequent changes of fresh autoclaved DI water every 12 h. The dialyzed solution was freeze‐dried for 3 days and the dried dSIS–NB was dissolved in sterile PBS using vigorous vortexing at 4 °C, stored at 4 °C, and used within 1 week. To ensure reproducibility, the gelation and mechanical properties of each batch were confirmed to fall within an acceptable range before use for further experiments.

### Proteomic Analysis of dSIS and dSIS–NB

Proteomic analysis was performed by Indiana Clinical and Translational Sciences Institute (CTSI) Center for Proteome Analysis. Briefly, 5 mg of lyophilized dSIS and dSIS–NB samples was resuspended in 40% acetonitrile at 2–4 µg µL^−1^. 8 m urea (30 µL) was added to 10 µL of dSIS/dSIS–NB sample (≈50 µg). Disulfide bonds were reduced with 15 mm tris(2‐carboxyethyl)phosphine (TCEP) and alkylated with 50 mm chloroacetic acid (CAA). The samples were digested overnight with 1 µg trypsin/LysC—(1 h at ≈6 m urea, then diluted to 2 m urea with Tris and added 2 µL PNGase F, which was allowed to go overnight at 35 °C). The next morning, samples were quenched with formic acid and cleaned up with Waters Sep‐Pak cartridge. Next, samples were resuspended in 40 µL of 0.1% formic acid (FA) and analyzed by the Eclipse‐Aurora column with FAIMS (CID), and analyzed in PEAKS 12 with custom modification of dSIS–NB.

### Synthesis of PEGNB‐T

PEGNB‐T was synthesized by reacting tyramine with the carboxylic acid on PEGNB_CA_, which was synthesized as previously reported.^[^ ^21^ ^]^ Briefly, 1 g of 8‐arm PEG─OH (0.4 mmol hydroxyl group), 0.328 g carbic anhydride (2 mmol, 5 equiv. to ─OH), 49 mg 4‐dimethylaminopyridine (DMAP, Sigma–Aldrich) (0.4 mmol, 1 equiv. to ─OH), and 6.64 mL of anhydrous tetrahydrofuran (THF, Sigma–Aldrich) were charged in a round bottom flask. The flask was placed in a 60 °C oil bath with constant stirring for 8 h. An additional 5 equiv. of CA and 1 equivalent DMAP were added to the flask and the reaction was continued for 24 h. The product was precipitated using 20× cold diethyl ether twice, redissolving in minimal dichloromethane (DCM, Fisher Scientific) between precipitations, and dried in a vacuum overnight before dialyzing for 72 h against ddH_2_O. After dialysis, the product, PEGNB_CA_, was lyophilized for 72 h under 20 Pa at −50 °C.

Next, tyramine was conjugated to 1 g of PEGNB_CA_ (carboxylic group: 380 µmol) using the standard carbodiimide chemistry. Briefly, 148.8 µL of *N*,*N*′‐diisopropylcarbodiimide (DIC, Chem‐Impex) (950 µmol, 2.5 equiv.) and 160 mg hydroxybenzotriazole (HOBt, Oakwood Chemical) (950 µmol, 2.5 equiv.) were mixed in 10 mL amine‐free anhydrous dimethylformamide (DMF, Fisher Scientific) and blanketed with nitrogen and stirred for 2 h in the dark. 165 mg tyramine hydrochloride (Chem‐Impex) (950 µmol, 2.5 equiv.), 165.4 µL *N*,*N*′‐diisopropylethylamine (DIPEA, TCI Chemicals) (950 µmol, 2.5 equiv.), and 23 mg DMAP (190 µmol, 0.5 equiv.) were dissolved in 1 mL of amine‐free DMF and added to the reaction. The mixture was allowed to react for 16 h, followed by dialysis in methanol for 1 day and in ddH_2_O for 2 more days, then lyophilized. The norbornene and tyramine substitution were characterized by 1H NMR spectra in D_2_O (Bruker 400 MHz).

### Crosslinking and Mechanical Characterization of Bulk dSIS–NB Hydrogels

dSIS–NB hydrogels were crosslinked by thiol–norbornene photoclick reaction. To prepare the hydrogel samples, 40 µL of the precursor solution (1.2 wt% dSIS–NB, various contents of PEG4SH, and 7 mm of photoinitiator LAP) was dispensed in between two glass slides separated with 0.8 mm Teflon spacers and pretreated with a water‐repellent coating. The assembly was placed under 365 nm UV light at 5 mW cm^−2^ for 2 min to yield dSIS–NB hydrogels with an approximate diameter of 8 mm and a thickness of 0.8 mm. Bulk hydrogel moduli were characterized by a modular rheometer (MCR 102, Anton Paar) operating in a strain–sweep mode with normal force (NF) control. The rheometrical testing was conducted at 25 °C, with a normal force of 0.25 N, shear strain from 1% to 5%, and a frequency of 1 Hz. The shear modulus (*G*′) in the linear viscoelastic region (LVE) was reported as the stiffness of the hydrogels.

### Cell Maintenance and Culture on Hydrogels

Caco‐2 (ECACC 86010202) and HT29‐MTX (ECACC 12040401) cell lines were maintained in 100 mm tissue culture dish (229621, Celltreat) in high‐glucose Dulbecco’s Modified Eagle Medium (DMEM) (SH30243.01, Cytiva) containing 10% fetal bovine serum (Gibco), 1% v/v nonessential amino acid (Fisher Scientific), and 1% v/v penicillin/streptomycin (Fisher Scientific) at 37 °C, 5% CO_2_ and 95% humidity. Cells were passaged at 80% confluence using 0.05% Trypsin ethylenediaminetetraacetic acid (Fisher Scientific) and the culture media were refreshed every 2–3 days. Both Caco‐2 and HT29‐MTX cells at passages 5–12 (passage 1: the first subculture after thawing the acquired frozen stocks) were used for the experiments. Cells at passages 1–5 were maintained in cryogenic storage in liquid nitrogen.

90 mL of dSIS–NB precursor (1.2 wt% dSIS–NB, 1.2 wt% PEG4SH, 7 mm LAP) or Matrigel (354230, Corning) were prepared in 24‐well glass bottoms plates (229125, Celltreat) for time‐lapse imaging, or in 24‐well plates with culture inserts (PI8P01250, Millipore) for all other experiments. The hydrogels were polymerized under 365 nm light (5 mW cm^−2^) for 2 min when using dSIS–NB or incubated for 30 min at 37 °C when using Matrigel. Caco‐2 and HT29‐MTX cells were trypsinized, pelleted, and aliquoted. The cells were applied by seeding 1 × 10^5^ cells cm^−2^ onto the surface of solidified hydrogels either mono or in co‐culture with ratio of 9:1 (Caco‐2:HT29‐MTX) as recommended from previous study.^[^ ^27^ ^]^ Subsequently, the plate was incubated for at least 1 h to allow the cells to attach and maintained at 37 °C, 5% CO_2,_ and 95% humidity. Medium was refreshed every 2–3 days and cultured over 21 days.

### Time‐Lapse Imaging and Analysis

Time‐lapse imaging was conducted using an automatic CELLCYTE X microscope (CELLINK AB) in a humidified, heated, CO_2_‐controlled chamber (VWR). Live cell imaging started 3 h after cell seeding, using a 4× objective. Pictures of the same positions were captured every 3 h over a recording duration from cell seeding to the confluence of cell monolayer. Four positions on each well were monitored, and the experiment was repeated on three wells for each hydrogel condition. Images were analyzed to determine cell confluence and videos were compiled using the tools integrated into CellCyte Studio.

### Fabrication of dSIS–NB Hydrogels with Crypt/Villus Topography

The overall process of creating an engineered human intestinal model is graphically described in Figure 2. A DLP 3D printer (LumenX, CELLINK) was used to create PEGNB‐T SHM with negative crypt/villus structures. The PEGNB‐T SHMs were crosslinked by thiol–norbornene photo‐click reaction at 405 nm. Bioink formulation and printing parameters (light intensity and exposure time) were obtained from the previous studies.^[^ ^34^ ^]^ PEGNB‐T SHM were printed with 4 wt% PEGNB‐T, 3 wt% PEG4SH, 1.5 mm tartrazine, and 10 mm LAP. 405 nm light (34 mW cm^−2^) was turned on for 9.5 s for each layer of printing. Printing fidelity was defined as the difference in the upper diameter of the wells and the tip diameter of the pillars between designed and printed structures

(1) $$\mathrm{Fidelity}\left(\%\right)=1-\frac{\mathrm{Designed}\;\mathrm{diameter}-\mathrm{printed}\;\mathrm{diameter}}{\mathrm{Designed}\;\mathrm{diameter}}\times 100\%$$

SHM was used to cast 40 µL of dSIS–NB precursor (1.2 wt% dSIS–NB, 1.2 wt% PEG4SH, 7 mm LAP), followed by exposure to 365 nm light at 5 mW cm^−2^ for 1 min. The cast hydrogel assembly (i.e., SHM + dSIS–NB hydrogels) was placed upside down in a transwell insert (PI8P01250, Millipore). The insert was precoated with 50 µL of the dSIS–NB precursor followed by another 365 nm light exposure for 1 min to secure the hydrogel assembly in the transwell insert. After photocrosslinking, 1× PBS was added to the transwell insert, allowing hydrolytic degradation of the SHM at 37 °C. After the autonomous removal of PEGNB‐T SHM, the crypt/villus structure was rinsed twice with PBS, followed by Caco‐2 and HT29‐MTX cells seeding 1 × 10^5^ cells cm^−2^ on the top of the crypt/villus structure. Cells were allowed to settle at 37 °C, 5% CO_2_ for 1 h before additional medium was added and incubated at 37 °C, 5% CO_2_.

### Gene Expression Analysis

After 7, 12, and 21 days of culture on either dSIS–NB hydrogels or Matrigel, Caco‐2 cells were collected for gene expression by reverse transcription–quantitative polymerase chain reaction (RT‐qPCR). Total RNA was isolated from the collected cells using NucleoSpin RNA kit (MACHEREY‐NAGEL), followed by complementary DNA (cDNA) generation with PrimeScript RT reagent kit (Takara, Cat# RR037A). RT‐qPCR was performed with TB Green Premix Ex TaqII kit (Takara, Cat# RR820L) for genes commonly selected for intestinal epithelial assessment (e.g., Villin (*VIL1*), mucus (*MUC2*), intestinal alkaline phosphatase (*ALPI*), proliferation marker (*CCND1*), multidrug resistance transporter (*MDR1*), and transporter gene (*SLC15A*)). The primer sequences are listed in Table S1 (Supporting Information). To adjust for variations in the cDNA synthesis, gene expression data were normalized to housekeeping genes (*GAPDH*) and analyzed statistically to compare differences between time points and between hydrogel conditions. Fold changes were calculated by the 2^−ΔΔ^ *^Ct^* method. Three independent replicates in each condition were performed.

### Immunofluorescence Staining

Cells cultured on 3D crypt/villus hydrogel models or 2D flat hydrogels were fixed with 4% w/v paraformaldehyde (Fisher Scientific) in PBS (Life Tech No. 20012068) for an hour at room temperature or 24 h at 4 °C, washed twice for 5 min with PBS, permeabilized with 0.3% Triton X‐100 (Sigma # T8787) in PBS for 10 min, and blocked with 3% bovine serum albumin (BSA) (Sigma # A2153) in PBS with 0.05% v/v Tween‐20 (Sigma–Aldrich) for 1 h. Subsequently, cells were incubated with primary antibodies: Vinculin, Paxillin, Ki67, mucus‐2 (MUC2), ZO‐1, CD326 (EpCAM), and E‐Cad, at 4 °C overnight, followed by washing with PBS (three times × 15 min). After incubating the corresponding secondary antibodies together with Alexa Fluor 647‐conjugated phalloidin (1:100) (Cytoskeleton/F‐actin) for 2 h at room temperature, cell nuclei were counterstained with DAPI (1:200) for 30 min. The antibodies used are detailed in Table S2 (Supporting Information). During staining, the entire device was covered with aluminum foil to reduce the fluorescence decrease due to photobleaching. Fluorescence images were taken with a bench‐top confocal microscope (BC43, Oxford Instrument). All immunostainings were repeated at least three times and representative images were shown.

### TEER Measurement

To assess the growth and integrity of epithelial barriers on flat or 3D crypt/villus models, TEER was measured every 3 days using an EVOM2 epithelial voltohmmeter with an STX03 electrode (World Precision Instruments). TEER measurements were taken over 22 days, including insert only (no hydrogel and no cells), insert + flat hydrogel (no cells), and insert + crypt/villus hydrogel (no cells) to set the initial blank values for the resistance of transwell membrane insert and hydrogel, which were subtracted from the TEER values of the experimental groups. Note that the hydrogel layer alone contributed minimally to the TEER values, while the resistance of membrane in culture medium was around 181 ± 8 Ω. Two independent replicates were conducted for the conditions without significant TEER increases, and four independent replicates were conducted for the conditions with significant TEER increases. Raw resistance data were converted into TEER values using the following equation:

(2) $$\mathrm{TEER}\;\left(\Omega \;cm^{2}\right)=\left(R_{M}-R_{C}\right)\times \mathrm{Membrane}\;\mathrm{area}$$

where *R*_M_ represents the resistance value (Ω) of the measured model with cells, *R*_C_ is the resistance value (Ω) of the blank control group, and *A* is the membrane area of the transwell insert (0.6 cm^2^, PI8P01250, Millipore).

### Absorption/Barrier Integrity Assay

The barrier integrity of intestinal epithelium on 2D flat and 3D crypt/villus topography was evaluated by assessing paracellular transport. One day before starting the experiment, cells were rinsed with PBS and incubated with fresh DMEM without phenol red. To assess the distribution of dextrans within the hydrogel, representative conditions were selected. A total of 400 µL fresh medium was added to the basolateral side, and 400 µL of the dextran mixture (4.4k‐TD (Sigma, T1037) and 500k‐FD (Sigma, FD500S)) was applied to the apical side at 0.1 mg mL^−1^ in medium. After 60 min of incubation at 37 °C, the solutions were removed, and a bench‐top confocal microscopy was used to observe dextran distribution within the dSIS–NB hydrogel.

For permeability measurements, only 4.4k‐TD was used. The medium was changed with 400 µL fresh medium in the basolateral side and 400 µL of 4.4k‐TD (0.1 mg mL^−^1) on the apical side. Cell‐free hydrogels with identical crypt/villus geometry were used as controls. Samples (100 µL) were collected from the basolateral side every 30 min for 3 h into a black 96‐well microplate (Greiner bio‐one, 655076), and the same volume of fresh medium was replenished after each sampling into basolateral side. Fluorescence intensity was measured using a SpectraMax iD5 Microplate Reader (Molecular Devices, CA, USA) with excitation/emission at 530/590 nm. Fluorescence intensities were normalized to initial basolateral concentrations. The samples were correlated to a standard curve. The total amount (including the amount in 100 µL of took out samples) of compound determined at each time point was used to calculate the apparent permeability of the compound (from apical to basolateral side) using the following equation:

(3) $$P_{\mathrm{app}}\;{=}^{\frac{dQ}{dt}}\;/\;\left(A\;\times \;C_{0}\right)$$

where d*Q*/d*t* is the amount of compound (mg) passing through the intestinal barrier per unit time (s), *A* is the surface area (cm^2^), *C*_0_ is the initial concentration of the compound (mg mL^−1^). Experiments were performed with three replicates.

Drug‐Induced Barrier Disruption Study The disruption of barrier integrity under drug‐induced conditions was evaluated by exposing models to increasing concentrations of staurosporine (i.e., 0, 0.5, 5, and 50 µm) in medium. Controls were treated with medium containing only 0.01% DMSO (Sigma, D8418). TEER measurements were taken prior to staurosporine exposure as a baseline and monitored hourly for the first 12 h, and then again from 21 to 24 h post exposure. Between each interval, the models were placed back into the incubator. TEER values were normalized to the TEER measured before staurosporine exposure. The dextran distribution with 500k‐TD and 4.4k‐TD was conducted after 24 h of exposure using the same method described for the absorption/barrier integrity assay.

The barrier disruption at 5 µm staurosporine was further analyzed by performing a leakage assay at 0, 9, and 24 h of exposure. 400 mL of 4.4k‐TD or 500k‐FD (0.1 mg mL^−1^) containing 5 µm staurosporine in medium was added separately to the apical side following the replacement of the basal medium with 400 µL of fresh medium. At each time point, 100 µL of aliquots was sampled at the basolateral side and then replaced with 100 µL of fresh medium, fluorescence intensity with excitation/emission at 485/535 nm for FITC‐dextran and 530/590 for TRITC‐dextran. The normalized fluorescence intensity and *P*_app_ values were calculated as described above. These experiments were carried out across three replicates.

### Intestinal Disease Modeling Using the “Pizza” Scaffold

The process of creating the “Pizza” scaffold followed the same fabrication method used for molded dSIS–NB hydrogels with crypt/villus topography. The modification involved a CAD design that incorporated both flat surfaces and crypt/villus topography, as shown in Figure 7b. Then, a mixture of Caco‐2 and HT29‐MTX cells were seeded onto pizza dSIS–NB scaffold to form a functional epithelium layer. The distribution of dextrans within the hydrogel was assessed using the previously described method. Confocal images were analyzed to quantify the fluorescent intensity of 4.4k‐TD and 500k‐FD transported along the distance from flat region through transition zone to crypt/villus zone using FIJI‐ImageJ.^[^ ^49^ ^]^

### Statistical Analysis

Statistical analyses were performed using GraphPad Prism 9.0 (GraphPad, La Jolla, USA). Relevant statistical methods were provided in the figure captions. All experiments were conducted with at least three independent repeats. Averaged values were expressed as mean ± SEM. Statistical significance was assumed for *p* < 0.05.

## Conflict of Interest

The authors declare no conflict of interest.

## Author Contributions

N.H.L. and V.T.D. contributed equally to this work. C.‐C.L. and N.H.L. conceived the idea and designed the research. N.H.L. performed the experiments and analyzed data. V.T.D. synthesized dSIS–NB and printed 3D scaffolds. J.B.B. prepared PEGNB‐T hydrogel and analyzed crosslinking and mechanical characterization of PEGNB‐T. N.H.L. and V.T.D. drafted the manuscript. C.‐C.L. supervised the project and edited the manuscript. All authors reviewed the data and final manuscript.

## Supporting information



Supporting Information

Supplemental Video 1

Supplemental Video 2

Supplemental Video 3

Supplemental Video 4

Supplemental Video 5

Supplemental Video 6

Supplemental Video 7

Supplemental Video 8

Supplemental Video 9

Supporting Information

## Data Availability

The data that support the findings of this study are available in the Supporting Information of this article.
