# Supplementary material for: Engineered Decellularized Matrix Hydrogels with Crypt–Villus Topography for Forming Functional Intestinal Epithelium
Source: Small. 2025 Oct 22;21(49):e06632. doi: 10.1002/smll.202506632 (PMC12696786; doi:10.1002/smll.202506632)
Supplement: Supplementary file 1 — Supporting Information [file SMLL-21-e06632-s001.docx]

Supporting Information for

**Engineered decellularized matrix hydrogels with crypt-villus topography for rapid formation of functional intestinal epithelium**

Ngoc Ha Luong,^#^ Van Thuy Duong,^#^ Jonathan B. Bryan, Chien-Chi Lin*

Weldon School of Biomedical Engineering, Purdue University, West Lafayette, IN. 47907

^#^These authors contributed equally to the work.

*Corresponding Author

Chien-Chi Lin, Ph.D.

Professor, Weldon School of Biomedical Engineering

Purdue University

206 Martin Jischke Dr. West Lafayette, IN, 47907

Contact Information:

Phone: (765) 495-7791

Emails: lin711@purdue.edu

**This file contains:**

Supplementary Methods

Supplementary Videos 1-9 Legends

Supplementary Figure S1-S12

**Other supporting materials for this manuscript include the following:**

Supplementary Videos 1 to 9

**Gene expression analysis**

**Table S1**. The primer sequences for qPCR used in this study.

| **Gene** | **Forward primer sequence (5’–3’)** | **Reverse primer sequence (5’–3’)** |
| --- | --- | --- |
| *GAPDH* | GCCTCCTGAAAAGAGAGTGGAAG | GCCTCCTGAAAAGAGAGTGGAAG |
| *ALPI* | CCAGGACATCGCCACTCAGC | CTCAGTGCGGTTCCACACATAC |
| *VIL1* | TGCTATCTATGGTGTGGGAAGG | TCCTGTAGTCTCTTGGTGTTGG |
| *MDR1* | GCCAAAGCCAAAATATCAGC | TTCCAATGTGTTCGGCAT |
| *CCND1* | CAATGACCCCGCACGATTTC | CATGGAGGGCGGATTGGAA |
| *SLC15A* | TGTCCACCGCCATCTACCATA | CCACGAGTCGGCGATAAGAG |
| *TJP-1* | ACCAGTAAGTCGTCCTGATCC | TCGGCCAAATCTTCTCACTCC |

**Immunofluorescence staining**

**Table S2**. List of antibodies used in study.

| **Antibody** | **Dilution** | **Source/Isotype** | **Supplier (Catalog #)** |
| --- | --- | --- | --- |
| **Primary antibodies** |  |  |  |
| E-Cadherin | 1:200 | Rabbit | Cell Signaling (3195) |
| EpCam (CD326) | 1:100 | Mouse | Thermo Scientific (14-9326-82) |
| Vinculin | 1:50 | Mouse | Santa Cruz Biotech. (sc-25336) |
| Paxillin | 1:50 | Rabbit | Abcam (ab32084) |
| ZO-1 | 1:100 | Rabbit | Thermo Scientific (61-7300) |
| Mucin 2/MUC2 | 1:100 | Mouse | Santa Cruz Biotech. (sc-515032) |
| Ki67 | 1:200 | Rabbit | Cell Signaling (9129) |
| Villin | 1:200 | Mouse | Thermo Scientific ([MA5-38658](https://www.thermofisher.com/order/genome-database/details/antibody/MA538658)) |
| **Secondary antibodies** |  |  |  |
| Anti-Rabbit Alexa Fluor 488 | 1:200 | Donkey | Invitrogen (A21206) |
| Anti-Mouse Alexa Fluor 488 | 1:200 | Goat | BioLegend (405319) |
| Anti-Rabbit Alexa Fluor 555 | 1:200 | Goat | Cell Signaling (4413S) |
| Anti-Mouse Alexa Fluor 555 | 1:200 | Goat | Cell Signaling (4409S) |

**Supplementary Video Legends**

**Video S1.** Time-lapse recording of Caco-2 cells cultured on a thick and flat dSIS-NB hydrogel over time (3-hr intervals for a total period of 294 hr). Scale bar: 100 μm.

**Video S2.** Time-lapse recording of Caco-2 cells cultured on a thick and flat Matrigel layer over time (3-hr intervals for a total period of 294 hr). Scale bar: 100 μm.

**Video S3.** 3D view of an immunofluorescence image for Caco-2 monolayer on a flat dSIS-NB hydrogel. Caco-2 cells were stained with E-Cadherin (green) and F-Actin (white). Nuclei were counter-stained with DAPI (blue). Scale bar: 100 μm.

**Video S4.** 3D view of an immunofluorescence image for Caco-2 clusters formation on a thick Matrigel layer. Caco-2 cells were stained with E-Cadherin (green) and F-Actin (white). Nuclei were counter-stained with DAPI (blue). Scale bar: 50 μm.

**Video S5.** 3D confocal fluorescence image of DLP printed sacrificial PEGNB-T hydrogel mold. The mold was conjugated with rhodamine-PEG-thiol (Rh-PEG-SH) for visualization. Scale bar: 200 μm.

**Video S6.** 3D confocal fluorescence image of inverse molded dSIS-NB hydrogel with intact crypt/villus structure after rapid dissolution of the sacrificial PEGNB-T hydrogel. The dSIS-NB crypt/villus hydrogel was conjugated with rhodamine-PEG-thiol (Rh-PEG-SH) for visualization. Scale bar: 200 μm.

**Video S7.** Time-lapse recording of Caco-2/HT29-MTX cell proliferation on dSIS-NB hydrogel with crypt-villus structure over time (3-hr intervals for a total period of 90 hr). Scale bar: 100 μm.

**Video S8.** 3D view of an immunofluorescence image for Caco-2/HT29-MTX co-culture on crypt/villus structure on day 1, day 2, and day 3. The cells were stained for F-actin (white) and nuclei were counter-stained with DAPI (blue). Scale bars for days 1 and 2: 150 μm. Scale bar for day 3: 200 μm.

**Video S9.** 3D view of an immunofluorescence image of a full cell coverage on crypt/villus structure on day 3. The models were stained for F-actin (white) and E-Cadherin (green). Scale bar: 200 μm.

**Supplementary Figures**

**Figure S1. Biochemical characterization of dSIS and dSIS-NB hydrogels**. Proteomic profiling of extracellular matrix proteins identified in dSIS and dSIS-NB, showing relative abundance of collagen types I–VI and fibrillins (FBN1, FBN2).

**
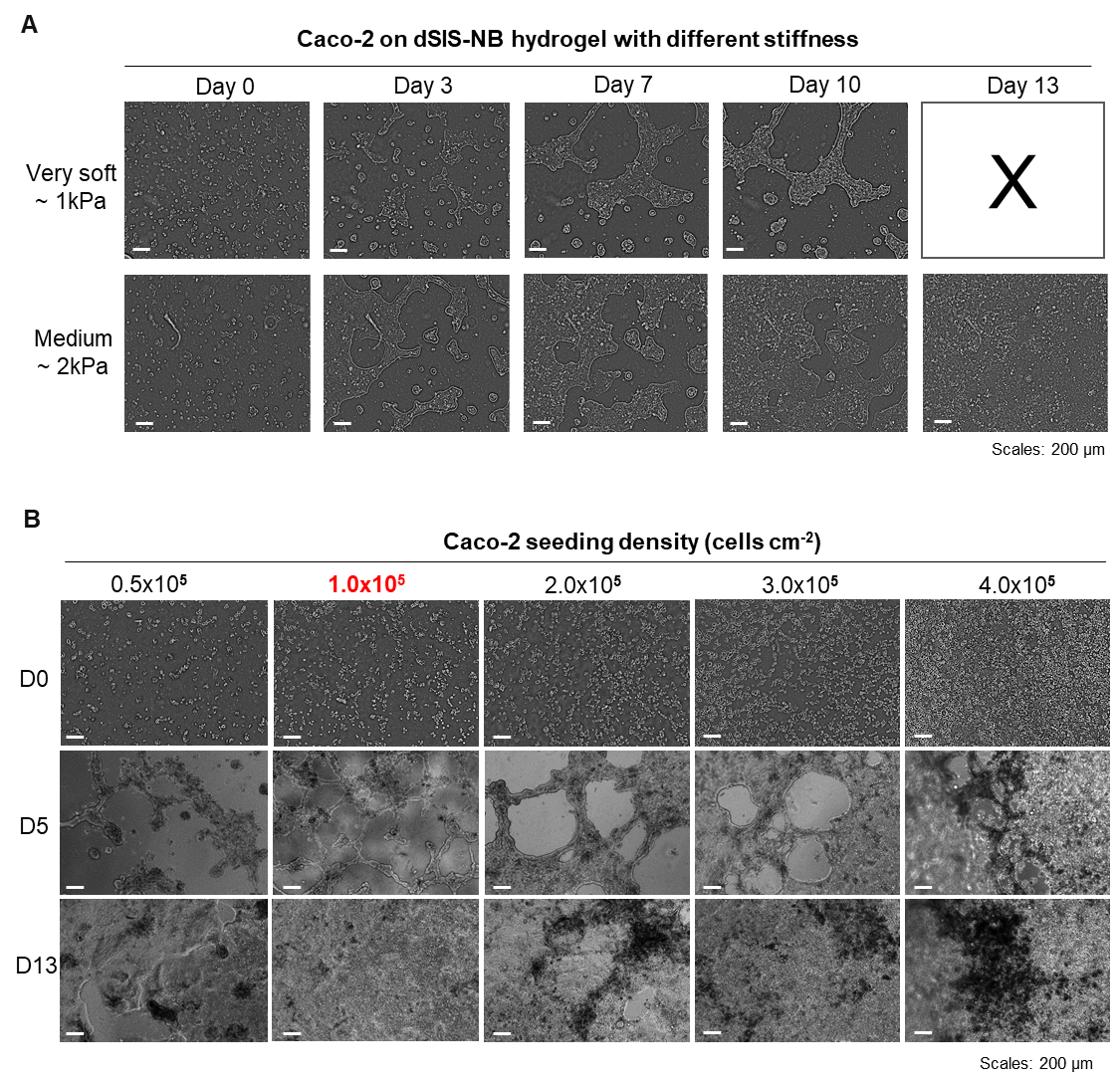
Figure S2**. **Effect of hydrogel stiffness and Caco-2 seeding density on the formation of cell monolayers on dSIS-NB hydrogel.** A) Snap-shot images of Caco-2 cells cultured on soft (~1kPa) and medium stiffness (~2 kPa) dSIS-NB hydrogel. The (X) image means the hydrogel was degraded and unable to support cells form a complete monolayer. B) Snap-shot images of Caco-2 cells cultured on dSIS-NB hydrogel (~2 kPa) at various seeding densities. The optimal seeding density (1x10^5^ cells cm^-2^, where the cells form a uniform, well-organized monolayer, is highlighted in **red**. At lower seeding densities, the confluence of the monolayer was delayed, leading to gaps in the monolayer and a less uniform structure. Conversely, at higher seeding densities, the cells accumulated unevenly, leading to overcrowding that did not achieve a uniform monolayer. Scale bars: 200 μm.

**
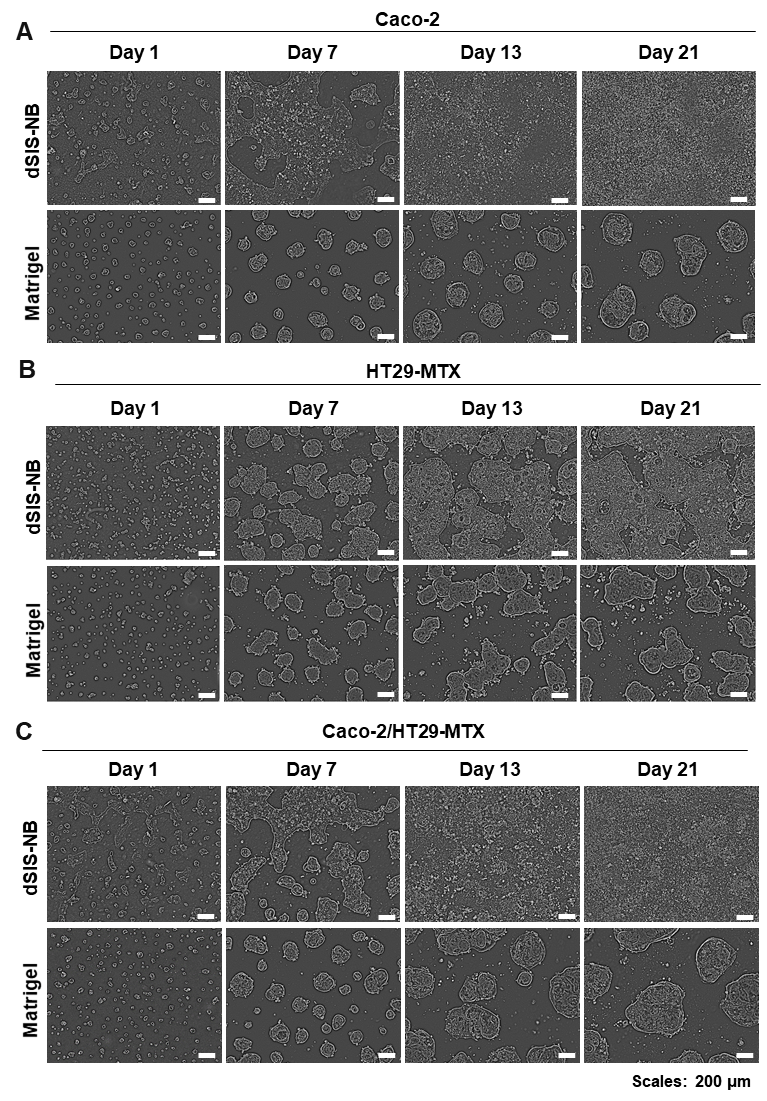
**

**Figure S3. Biocompatibility assessment based on intestinal cell behaviors on dSIS-NB hydrogels and Matrigel**. Representative time-lapse image sequences that compare the behavior of intestinal cell lines cultured on dSIS-NB hydrogel and Matrigel over time. The three conditions include: **A)** Caco-2 monoculture, **B)** HT29-MTX monoculture, **C)** Caco-2/HT29-MTX co-culture. Scale bar: 200 μm. The images demonstrated that dSIS-NB hydrogel supports the proliferation and organization of intestinal epithelial cells, both monocultures and co-cultures, more effectively than Matrigel.


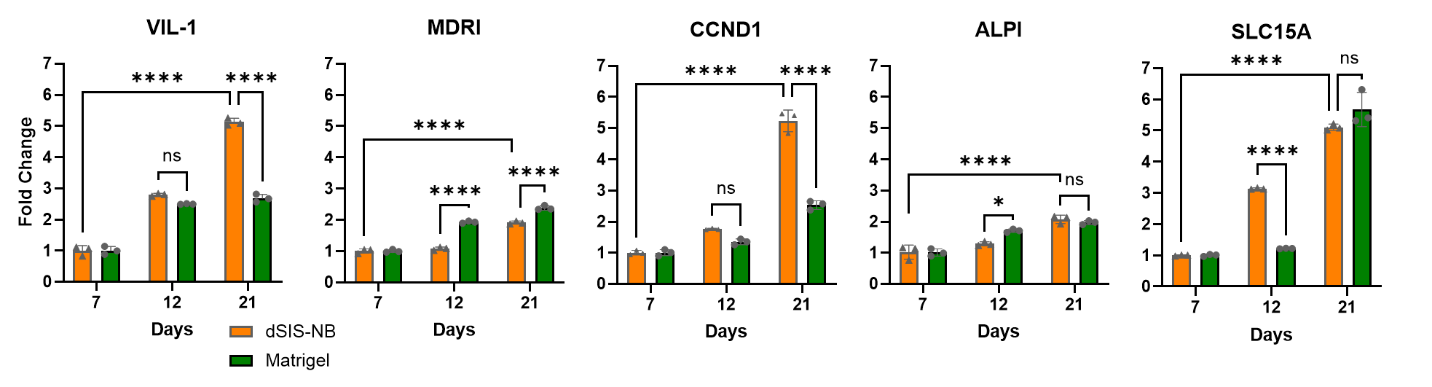


**Figure S4.** **Maturation of Caco-2 cells cultured on dSIS-NB hydrogel and Matrigel**. mRNA expression levels of intestinal epithelial markers in Caco-2 cells cultured on dSIS-NB hydrogels and Matrigel. VIL1: Villin, MDR1: multidrug resistance, CCND1: cell cycle marker, cyclin D1, ALPI: intestinal alkaline phosphatase, and SLC15A: transporter gene. Data are presented as mean ± SEM; (n = 3, ^*^*p* < 0.05, ^**^*p* < 0.01, ^***^*p* < 0.001, and ^****^*p* < 0.0001 by Two-way ANOVA Multiple Comparisons Tukey’s Post Hoc Test). The expression of these markers significantly increased on day 21 in Caco-2 cells cultured on both dSIS-NB hydrogel and Matrigel, reflecting enhanced epithelial differentiation and functional maturation.


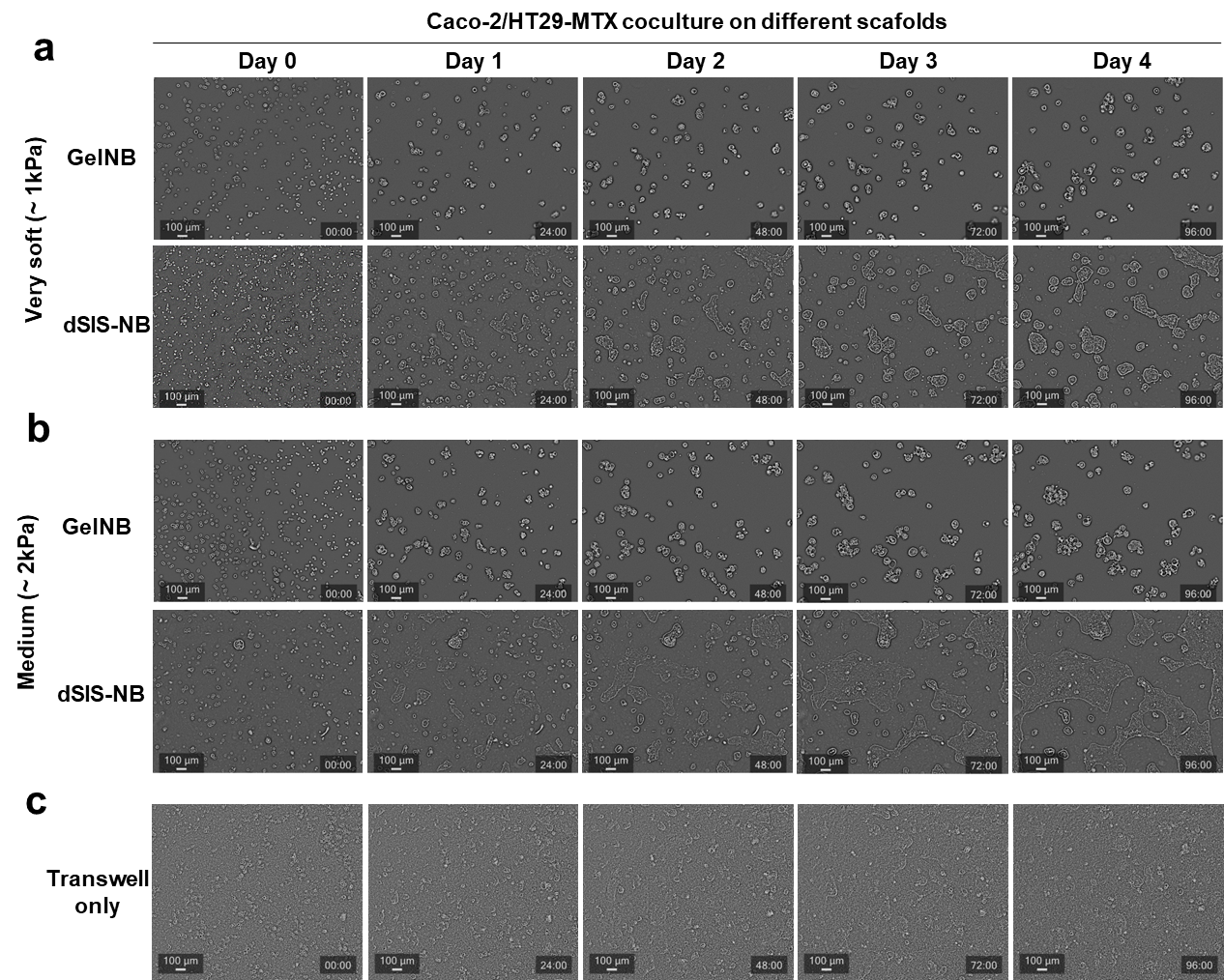


**Figure S5**. **Effect of hydrogel biochemical composition and stiffness on Caco-2/HT29-MTX cell behavior. a** and **b)** GelNB-PEG4SH hydrogels were used as a control for culturing Caco-2/HT29-MTX cells. GelNB at 5 wt% was crosslinked with PEG4SH at 0.9 wt% for a soft hydrogel with G” ~1 kPa or with 1.3 wt% PEG4SH for a medium stiffness hydrogel with G’ ~2 kPa. Caco-2/HT29-MTX cell adhesion and growth on dSIS-NB hydrogels with similar stiffness of 1 kPa to 2 kPa were shown for comparison. **c**) Transwell used as a control for conventional models.


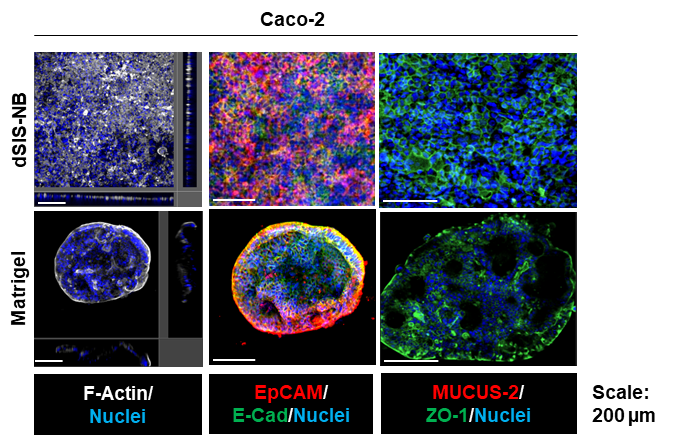


**Figure S6.** **Assessment of morphology and cell-to-cell interaction in Caco-2 monoculture on dSIS-NB hydrogels or Matrigel**. Immunostaining of key markers involved in cell morphology and cell-to-cell interactions in Caco-2 monoculture cultured on dSIS-NB hydrogels or Matrigel. F-actin (actin cytoskeleton), EpCAM (epithelial cell adhesion protein), E-Cad (E-cadherin, a cell-cell adhesion protein), Mucus-2 (mucin protein), and ZO-1 (tight junction protein). Mucus-2 was not detected in Caco-2 monocultures.


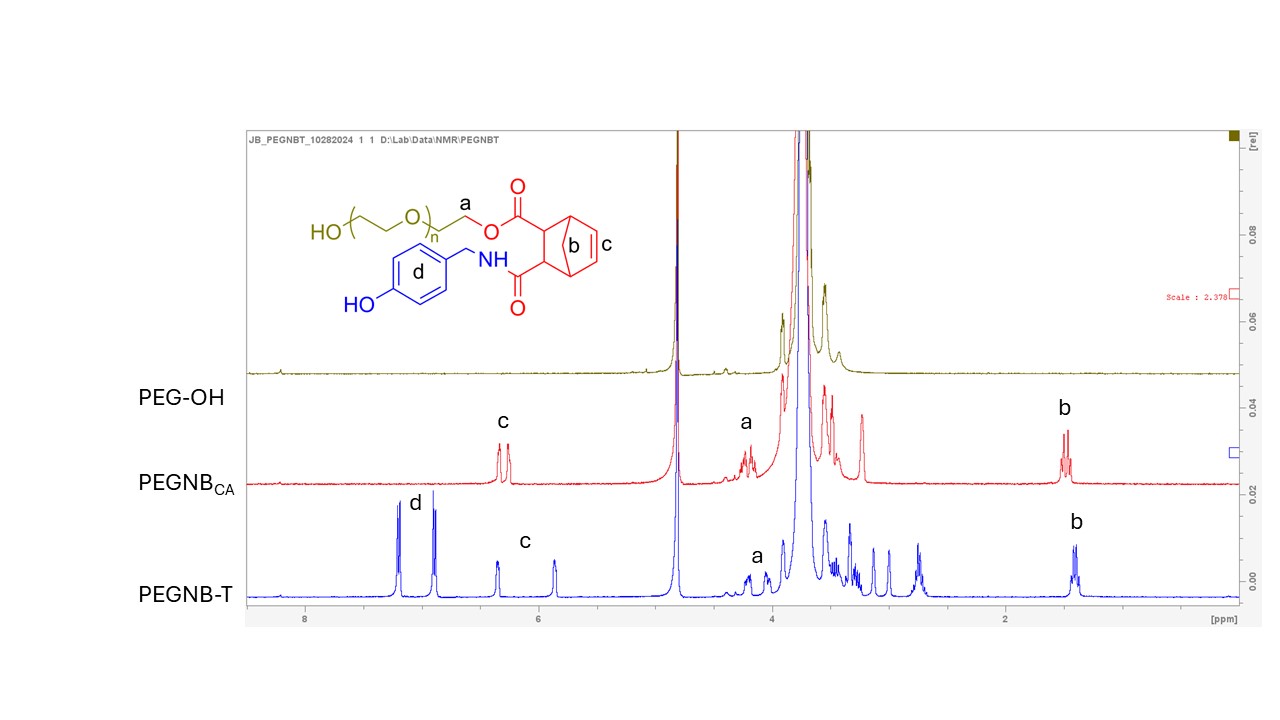


**Figure S7**. **1H NMR spectra of PEG-OH, PEGNB*CA*, and PEGNB-T**. The comparison of the proton nuclear magnetic resonance (1H NMR) spectra of three different polymers: PEG-OH (yellow), PEGNB*CA* (red), and PEGNB-T (blue). Peak a: PEG alkane protons, Peak b: norbornene alkane protons, Peaks c: norbornene alkene protons, and d: tyramine aromatic protons. PEG-OH: Shows the characteristic peaks of the polyethylene glycol backbone. PEGNB*CA*: The NMR spectrum of PEG functionalized with carbic anhydride clearly displays additional peaks for corresponding to the strained alkene (c, ~6.4 ppm) and the alkane protons (b, ~1.4). PEGNB-T: The spectrum of PEGNB-T exhibits the typical PEG backbone peak a (~ 4 ppm), norbornene peaks b (~1.4 ppm) and c (~6.4 ppm), as well as additional peaks for aromatic ring protons (d) in the range of 6.6-7.0 ppm, indicating successful conjugation of tyramine(T) to PEGNB.


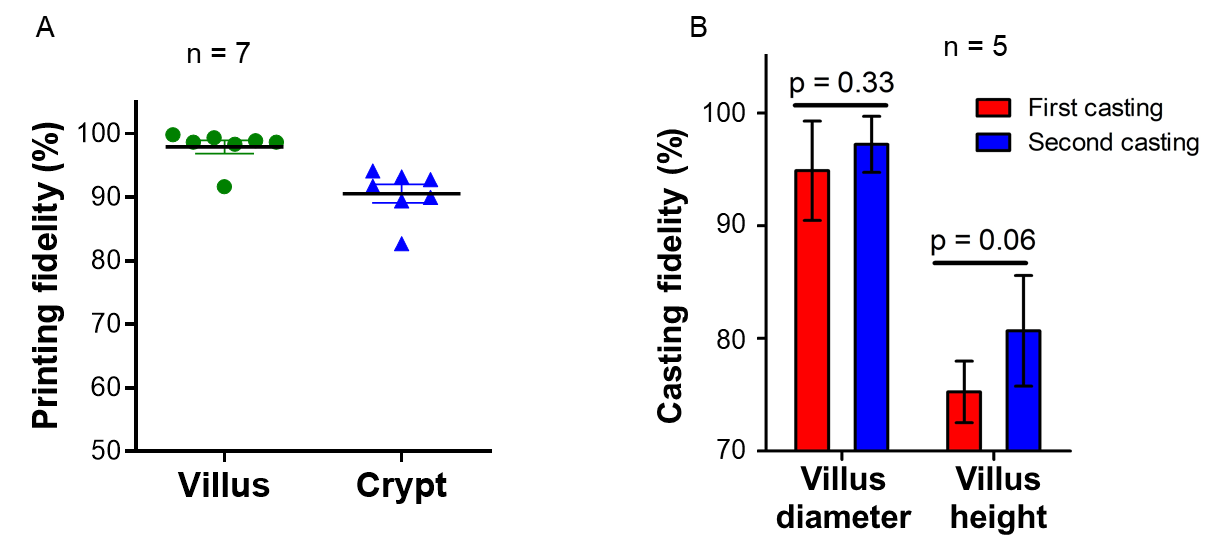


**Figure S8**. Fidelity of DLP-printed PEGNB-T mold and dSIS-NB villus/crypt topography. (A) Fidelity of DLP-printed PEGNB-T hydrogels with negative villus/crypt topography. Quantitative analysis was performed on seven samples (n=7), focusing on key geometrical parameters such as upper diameter of the wells and the tip diameter of the pillars. DLP printing achieved high fidelity in replicating the intricate topography, reflecting the precision of the photopolymerization process. (B) Fidelity of dSIS-NB villus/crypt topography was performed on five samples (n=5), focusing on key geometrical parameters such as base diameter and height of the villi. Molded dSIS-NB achieved high fidelity and consistent uniformity (*p* > 0.05, *t*-test) across the samples. The results confirmed the reproducibility of the sacrificial molding approach to fabricating the intricate crypt-villus topography on dSIS-NB hydrogel with high fidelity.

**Figure S9**. **Hydrolytic degradation of DLP-printed PEGNB-T hydrogels.** Tartrazine concentrations tested are **0 mM** (control, red diamonds), **0.75 mM** (blue triangles), and **1.5 mM** (green squares). The G’ of the hydrogels is measured upon fabrication, after 60 minutes and 120 minutes (n=3) to assess the impact of tartrazine on short-term hydrolytic degradation. Tartrazine did not affect significantly to the final hydrolytic degradation rate of PEGNB-T hydrogels after 120 min.


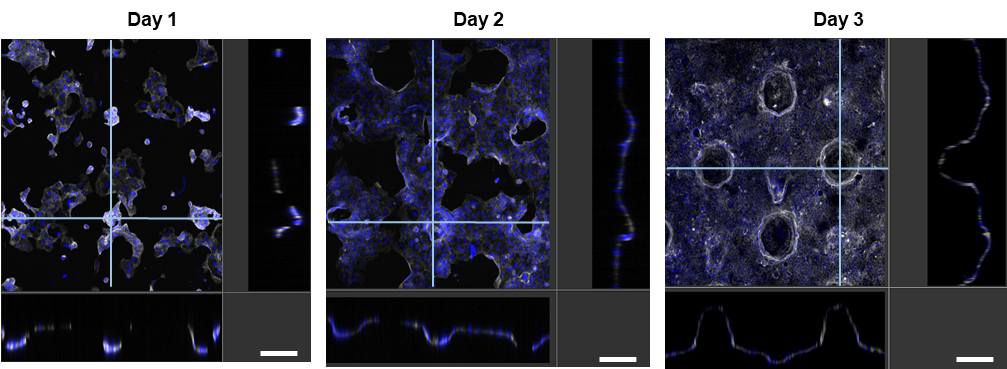
**Figure S10**. Formation of Caco-2/HT29-MTX cell monolayer on the crypt-villus dSIS-NB hydrogels for the first three days. Cell morphology and organization assessed using F-actin staining (white) and DAPI counterstaining for nuclei (blue). Scale bars: 200 μm. This early morphogenesis suggests that the crypt-villus architecture of dSIS-NB hydrogel promotes rapid and organized epithelial layer formation.


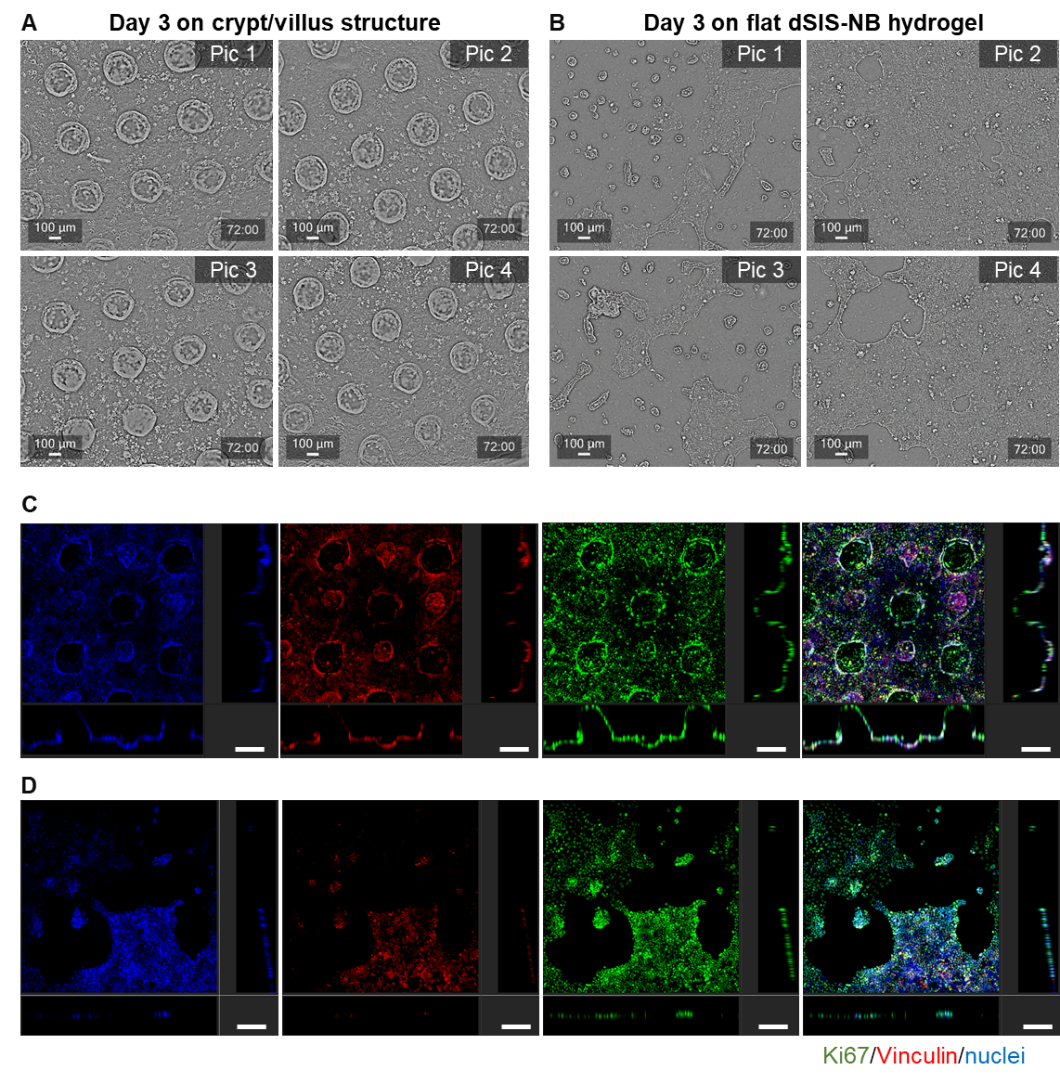


**Figure S11**. **Effects of gel surface topography (crypt/villus or flat) on monolayer formation**. (**A, B**) Bright-field images of selective areas on dSIS-NB hydrogels with (**A**) crypt/villus structure or (B) flat surface after 3 days of Caco-2/HT29-MTX cell culture. The crypt/villus topography (**A**) promoted more organized monolayer formation, while the flat surface (**B**) showed an uneven spreading. (**C, D**) Confocal z-stack images of Caco-2/HT29-MTX cells cultured on dSIS-NB hydrogels with (**C**) crypt/villus structure and (**D**) flat surface. Immunostaining for **Ki67** (green), a proliferation marker, and **Vinculin** (red), a focal adhesion protein, was performed to assess cell proliferation and adhesion on day 3. Cells cultured on the crypt/villus structure (**C**) exhibited evenly Ki67 expressionalong with well-defined Vinculin localization. In contrast, cells on the flat surface (**D**) showed uneven Ki67 expression and less defined Vinculin staining. Scale bars: 200 μm.

**
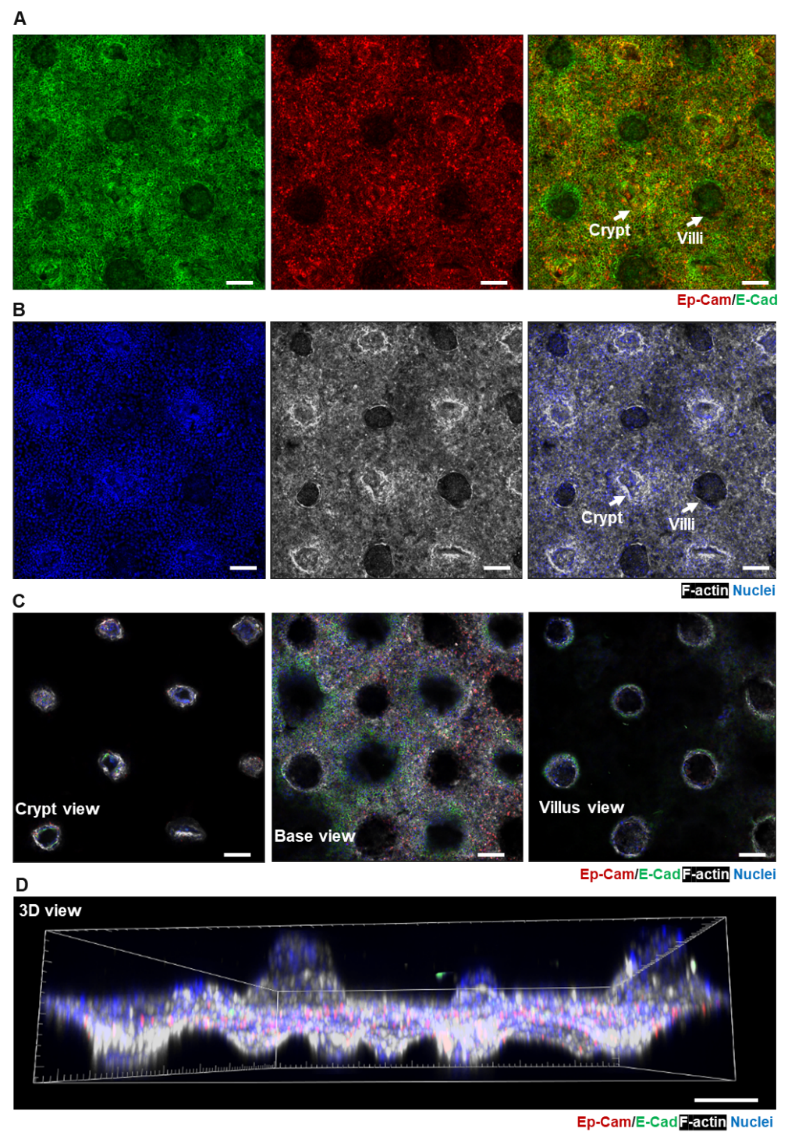
**

**Figure S12**. **Immunofluorescence staining for 3D crypt/villus structure after 21 days of culture**. (**A, B**) Confocal z-stack images of E-cad (green), EpCAM (red), F-actin (white), and nuclei (blue) staining of the Caco-2/HT29-MTX cell monolayer. The staining revealed prominent expressions of **E-cadherin** and **EpCAM (A)**, both of which are indicative of strong cell-cell adhesion, which was maintained throughout the culture period, suggesting a well-formed epithelial layer. The **F-actin (B)** staining highlights organized cytoskeletal networks, further supporting the well-structured monolayer. (**C, D**) Cross-sectional images of a single layer (crypts, villus base, and villi) and a 3D view obtained by merging all markers. The cross-sectional images (**C**) demonstrate the preservation of the crypt-villus architecture with well-defined crypts and villus structures, even after 21 days of culture. The 3D view (**D**) reinforces the integrity and stability of the crypt/villus morphology throughout the culture period. Scale bars: 200 μm.

**
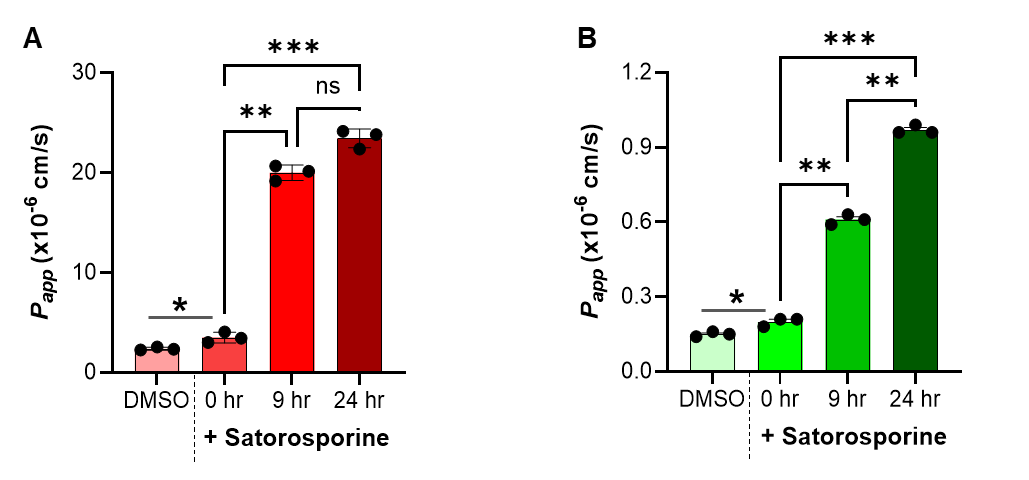
**

**Figure S13. Effect of Staurosporine (5 μM) on the permeability of (A) 4.4 kDa TRITC-dextran and (B) 500 kDa FITC-dextran.** Staurosporine (5 μM), a known inducer of apoptosis, was added at the start of the experiment to assess its effect on the barrier integrity of the Caco-2/HT29-MTX monolayer cultured on dSIS-NB hydrogels with crypt/villus structures. Permeability was measured by adding (**A**) 4.4k-TD and (**B**) 500k-FD at indicated time points (i.e. 0 hr, 9 hr, and 12 hr) after staurosporine treatment. The results show a significant increase in the permeability of both dextrans, indicating a loss of barrier function. The delayed and limited passage of 500 kDa dextran suggests that tight junctions, although compromised by staurosporine, still partially restricted the movement of larger molecules. Data are presented as mean ± SEM; (n = 3, ^*^*p* < 0.05, ^**^*p* < 0.01, ^***^*p* < 0.001, and ^****^*p* < 0.0001 by repeated measures one-way ANOVA).
